# ENZYMAP: Exploiting Protein Annotation for Modeling and Predicting EC Number Changes in UniProt/Swiss-Prot

**DOI:** 10.1371/journal.pone.0089162

**Published:** 2014-02-19

**Authors:** Sabrina de Azevedo Silveira, Raquel Cardoso de Melo-Minardi, Carlos Henrique da Silveira, Marcelo Matos Santoro, Wagner Meira Jr

**Affiliations:** 1 Department of Computer Science, Universidade Federal de Minas Gerais, Belo Horizonte, Minas Gerais, Brazil; 2 Department of Biochemistry and Immunology, Universidade Federal de Minas Gerais, Belo Horizonte, Minas Gerais, Brazil; 3 Department of Biotechnology, Universidade Federal da Paraíba, João Pessoa, Paraíba, Brazil; Memorial Sloan Kettering Cancer Center, United States of America

## Abstract

The volume and diversity of biological data are increasing at very high rates. Vast amounts of protein sequences and structures, protein and genetic interactions and phenotype studies have been produced. The majority of data generated by high-throughput devices is automatically annotated because manually annotating them is not possible. Thus, efficient and precise automatic annotation methods are required to ensure the quality and reliability of both the biological data and associated annotations. We proposed *ENZYM*atic *A*nnotation *P*redictor (ENZYMAP), a technique to characterize and predict EC number changes based on annotations from UniProt/Swiss-Prot using a supervised learning approach. We evaluated ENZYMAP experimentally, using test data sets from both UniProt/Swiss-Prot and UniProt/TrEMBL, and showed that predicting EC changes using selected types of annotation is possible. Finally, we compared ENZYMAP and DETECT with respect to their predictions and checked both against the UniProt/Swiss-Prot annotations. ENZYMAP was shown to be more accurate than DETECT, coming closer to the actual changes in UniProt/Swiss-Prot. Our proposal is intended to be an automatic complementary method (that can be used together with other techniques like the ones based on protein sequence and structure) that helps to improve the quality and reliability of enzyme annotations over time, suggesting possible corrections, anticipating annotation changes and propagating the implicit knowledge for the whole dataset.

## Introduction

In recent decades there has been a surge in the amount of biological data available. According to [1], new DNA sequencing technologies allowed a 1000-fold drop in sequencing costs since 1990 and made an increasing number of large data collection projects economically possible, leading to an exponential increase in the DNA sequence data available. Additionally, vast amounts of data, such as protein sequences and structures, gene-expression measurements, protein and genetic interactions and phenotype studies, have been generated [2]. A significant portion of these data are organized and publicly available to the scientific community in biological repositories and databases accessible through the Internet. In accordance with [3], these biological databases store not only biological raw data but also relevant information such as protein function, literature information and the relationship between a protein and its encoding gene, among other annotation.

Considering the existing and the increasing volumes of biological data, a common approach involves selected data sets of high relevance being manually curated by experts while most data are automatically annotated [4]. In the majority of cases, the roles of genes have been reported by sequence similarity propagation without experimental evidence [5], [6]. Glycoprotein G of the Nipah virus (entry Q9IH62 in Swiss-Prot) illustrates the drawbacks of this approach. When considering residue similarity, it is very similar (more than 50%) to hemagglutinin-neuraminidases, an enzyme group associated with viral attachment and fusion to the host cell. The structures of Glycoprotein G of the Hendra and Nipah viruses were solved (PDB id 2 VSK and 2 VSM, respectively), revealing the six-blade 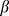 propeller structural motif typical of these hydrolases [7]. A structural alignment with a legitimate neuraminidase from Parainfluenza Virus Type III (PDB id 1 V3D), which also belongs to the same Paramyxoviridae family of Henipavirus, resulted in a RMSD lower than 2.0 Å [8]. Thus, an automated system based on such similarities may erroneously classify the function of Glycoprotein G of Henipavirus as having neuraminidase activity. In fact, up to release 14 (July 2008) of UniProt/Swiss-Prot [9], entry Q9IH62 was considered an enzyme. However, despite all these sequence and structural similarities, Henipavirus Glycoproteins G are now known not to be enzymes and to have only hemagglutinin activity, performing protein-protein interactions with host receptors [7]. At the time we wrote this article, the PDB [10] still classified them as hydrolases. In summary, the scientific community still has concerns regarding the quality and reliability of the data and annotations from the large, publicly available databases.

As we mentioned, biological repositories and databases almost always store some annotations that characterize and provide biological context to the raw data. In this work, we investigate the extent to which these annotations may be used to detect problems in the database. In particular, we want to verify whether the UniProt/Swiss-Prot annotations are good indicators that an EC number change (a type of enzyme annotation detailed in the next section) will occur and determine how we can systematically perform such predictions.

In this work, we propose a supervised learning approach to characterize and predict annotation changes in temporal data, which we named *ENZYM*atic *A*nnotation *P*redictor (ENZYMAP). More precisely, we are interested in predicting enzyme function annotation changes based on other UniProt/Swiss-Prot annotations. This proposal is intended to be an automatic complementary method (that can be used together with other techniques like the ones based on protein sequence and structure) that helps improve the quality and reliability of enzyme annotations, suggesting possible corrections and anticipating annotation changes. A common phenomenon in biological databases is that since a correction is made, this knowledge is not necessarily propagated to the whole database at once, but gradually and slowly. Our proposal can suggest corrections to database annotations, propagating the implicit knowledge for the whole dataset. Moreover, there is a huge volume of data that cannot be analyzed manually, hence the importance of reliable automatic annotation methods.

### Enzyme Annotations

In this work, enzyme function annotation refers to the Enzyme Commission (EC) number [11], which is a numerical classification scheme for enzymes based on the chemical reactions they catalyze. Each enzyme code consists of four numbers separated by periods. Those numbers represent a hierarchical, progressively finer classification of the catalyzed reaction. For example, the code *3.4.21.4* represents the following information: (*3*) hydrolase, indicating that the enzyme breaks a chemical bond involving a water molecule; (*3.4*) peptidase, indicating that the broken bond is a peptide bond, i.e., a bond between residues in a protein chain; (*3.4.21*) endopeptidase, indicating that an intra-chain peptide bond is broken and that a serine residue participates in the mechanism of catalysis; and (*3.4.21.4*) trypsin, indicating an enzyme that cleaves mainly at the carboxyl side of lysine or arginine residues.

The EC classification system is known to have some drawbacks. [12] reported a systematic annotation error in genome and pathway databases resulting from the misinterpretation of partial EC numbers. The key issue is that different enzymes that catalyze different reactions within the same class can be assigned the same partial EC number but the same partial EC number does not mean that the enzymes have the same activities. Also according to [12], the available EC number list does not cover all known enzymatic activities, so a partial EC number can be used even when the enzyme activity is known because a complete EC number is not available. [13] stated that the same reaction can be correctly annotated with different EC numbers. For example, the reaction catalyzed by the sterol 14-demethylase (1.14.13.70) is correctly assigned to 1.14.13 (oxidoreductase with NADH or NADPH as one donor and incorporation of one atom of oxygen), but it could be assigned to 1.14.21 (oxidoreductase with NADH or NADPH as one donor and the other dehydrogenated). These two sub-subclasses are similar and could be merged without a loss of information. In addition, according to [13], the general principle that the enzyme class is defined by its chemical reaction is violated in some cases. For example, the reaction 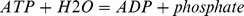 is catalyzed by the enzymes adenosinetriphosphatase (3.6.1.3) and myosin ATPase (3.6.4.1). In 3.6.1.3, the ATPase activity is not connected to actin movement, but in 3.6.4.1 it is.

Nonetheless, we chose to analyze the EC number as an enzyme function annotation because it is a mature and widely adopted enzyme classification scheme yet a controlled vocabulary that is numerical and hierarchical, which makes it particularly complex and interesting for computational modeling and description.

### Related Work

Several studies have drawn attention to the error rates in biological database annotation. Here, we briefly review some of them. In [6], authors compared annotations in *Mycoplasma genitalium* performed by three different groups and detected an error rate from 7% to 15% (depending on the gene analyzed and the group responsible for the analysis). [14] estimated the error rates in the genomes of *Mycoplasma genitalium*, *Haemophilus influenzae* and *Methanococcus jannaschii* by counting the number of discrepancies in sets of similar proteins and concluded that the error rates vary from 4% to 40% for the first genome and from 4% and 34% for the last two genomes. Both analyses were based on the discrepancies of annotations made by different research groups for very specific genomes, which allows the placement of a lower limit on the likely levels of misannotation according to [15].

A systematic annotation error in genome and pathway databases that results from the misinterpretation of partial EC numbers was reported in [12]. This error results in the assignment of genes annotated with a partial EC number to many or all biochemical reactions that are annotated with the same partial EC. For example, in KEGG [16], out of 135 genes from *Escherichia coli* annotated with a partial EC number, 58 were incorrectly assigned to reactions.

In [15], authors investigated the levels of misannotation for the molecular function in UniProtKB/Swiss-Prot, GenBank Non-redundant (NR) [17], UniProtKB/TrEMBL and KEGG for 37 enzyme families with experimental evidence. Swiss-Prot presented error levels close to 0% for most families, whereas GenBank NR, TrEMBL and KEGG showed high levels of misannotation, from 5%–63%, across the six studied superfamilies. Even in Swiss-Prot, a few families showed high levels of misannotation, for example Adenosine deaminase, which presented about 70% of misannotation. Furthermore, an analysis of the sequences from GenBank NR showed that the level of misannotation was close to 0% in 1999 but was approximately 40% in 2005, indicating that misannotation increased during that period.

The authors of [13] investigated inconsistencies in the EC number classification scheme as they can lead to inconsistencies in enzyme annotation. The authors validated the data of 3,788 enzymatic reactions and found a greater than 80% agreement between their assignment and the EC scheme. These results can be used to make corrections and improve the EC number classification.

These works focused on the levels of misannotation, showing that they are significant in a variety of databases, even those with manual revision such as UniProt/Swiss-Prot. The following works are related to annotation prediction tools and a comparison of computational methods for function prediction. The Density Estimation Tool for Enzyme ClassificaTion (DETECT), a probabilistic method for enzyme prediction based on both global and local sequence alignments, was presented in [18]. It uses a Bayesian framework to integrate information from density estimation profiles generated for each EC number. Compared with BLAST, DETECT improved the enzyme annotation accuracy and, when applied to *Plasmodium falciparum*, identified potential annotation errors.

In [19], the EnzymeDetector was implemented to automatically compare and evaluate the assigned enzyme functions from some annotation databases (NCBI RefSeq [20], KEGG, PEDANT [21], Pseudomonas Genome Database [22] and UniProt/Swiss-Prot) and to supplement them with its own function prediction. In the same work, the authors analyzed nine prokaryotic genomes and found approximately 70% inconsistencies in the enzyme predictions of the annotation resources used.

A system to provide annotations to proteins from completely sequenced microbial genomes was proposed in [23]. It is called HAMAP and is a semi-automated system that uses annotation templates manually built for protein families to propagate annotation to all members of manually defined protein families. This system has increased the speed at which microbial proteins are annotated in UniProt/Swiss-Prot without losses in the quality standards of this database.

Funtree is presented in [24] as a resource that combines structural, sequence, phylogenetic and functional data for structurally defined enzyme superfamilies. The authors stated that combining these data into a single resource enables the investigation of how novel enzyme functions have evolved, which can help to predict the functions of uncharacterized enzymes.

Recently, fifty-four methods for computational protein function prediction, which represent the state of the art, were evaluated on 866 proteins from 11 organisms in [25]. The two main findingns of this study were that the best algorithms used nowadays for function prediction are significantly better than first-generation methods and that although the top methods perform well to guide experiments, there is significant room to improve computational protein function prediction.

In this work, we propose ENZYMAP, a strategy based on supervised learning to characterize and predict EC number changes in temporal data from UniProt/Swiss-Prot using other types of annotation that are already available in the database. Our method is able to suggest possible corrections and anticipate annotation changes, which improves the quality and reliability of enzyme annotations. To the best of our knowledge, there are no other works that proposes this type of approach with such purpose.

## Materials and Methods

To characterize and predict EC number changes, we performed three types of supervised learning experiments in this work: *Descriptive Multiclass*, which is intended to verify whether separating entries in UniProt/Swiss-Prot that suffered a specific change in the EC number from those that remained constant based on entry annotation is possible; *Predictive Multiclass*, which attempts to use all available data in the database to predict an upcoming EC change; and *Predictive Common Source*, which segments EC changes by their common source (EC annotation before the change) to improve the latter experiment. In the next sections, we detail the data employed in these experiments, how the EC changes were modeled and the techniques used to construct our approach.

### Data

The EC number annotations of entries from the biological database UniProtKB/SwissProt were studied in this work. A set of 44 major releases available in UniProt web site [26] in May 2012 were downloaded. Releases 1 to 44 were analyzed.

To determine whether a specific UniProt/Swiss-Prot entry has undergone an EC number change, checking that entry's EC number in two consecutive releases is necessary, and therefore the 44 releases were analyzed in pairs, taking the set intersection of identifiers in two consecutive releases. A total of 18,727,155 EC pairs were obtained from the entire data set. Among them, 55,908 are pairs with different EC numbers.

The total number of entries, the number of entries annotated with an EC number and their percentage in the 44 releases are provided in Table S1 and Figure S1 in Material S1. The number of entries in the set intersection of each release pair is shown in Table S2 and Figure S2 in Material S1.

In addition to these data, we obtained the releases 43 and 44 from UniProt/TrEMBL (the latest releases by the time we started this work) to use as test data set. These data have a total of 21,570,363 EC pairs, from which 5,532 are pairs with different EC numbers.

#### Selected line types

In addition to the EC number change data from the 44 UniProt/Swiss-Prot releases, in the Descriptive Multiclass, Predictive Multiclass and Predictive Common Source Experiments, we are interested in entry line types able to characterize entries and their EC number changes. Next, we detail the selected line types and explain why they were chosen.

*Organism Classification* (OC), which refers to the taxonomic classification of the source organism. This classification is maintained by the National Center for Biotechnology Information (NCBI) [27] and reflects current phylogenetic knowledge. OC was selected because there are extensively studied and well annotated organisms which provide good training data for our supervised learning approach. These kind of data potentially lead to good quality annotation. *Saccharomyces cerevisiae*, *Drosophila melanogaster* and *Caenorhabditis elegans* are examples of well studied organisms. Althoug in general OC line type does not change over time (as it is submitted by researchers and identifies the organism in which a protein is present) the EC number associated with an entry can change and OC helps to characterize such entry;

*Reference Position* (RP) describes the extent of the work (reference) relevant to the entry and contains a description of the information propagated in such entry. Entries with more specific references in RP (e.g., function) likely have better annotation than entries with general references (e.g., large scale genomic DNA). So, RP characterize proteins by providing information about references used to annotate them.

*KeyWord* (KW), provides information that can be used to generate indexes of the sequence entries based on functional, structural, or other categories and represents a controlled vocabulary which summarises the content of an entry using relevant words related to that protein. Once again, it is able characterize proteins in Swiss-Prot, which is important for our supervised learning approach.

An example of the selected line types is provided below for UniProt/Swiss-Prot id P66880, whose EC number is currently 3.1.3.5. Further information regarding the line types OC, RP and KW from UniProt text file format can be obtained from the UniProt User Manual [28].

OC Bacteria; Proteobacteria; Alphaproteobacteria; Rhizobiales;

OC Brucellaceae; Brucella.

RP NUCLEOTIDE SEQUENCE [LARGE SCALE GENOMIC DNA].

KW Complete proteome; Cytoplasm; Hydrolase; Metal-binding;

KW Nucleotide-binding.

### Problem modeling

#### Initial exploration

Based on the numerical and hierarchical nature of the EC number, we proposed a model to characterize the EC changes observed across the releases of UniProt/Swiss-Prot. Knowing the hierarchical level in which a change occurs is important because an alteration at a higher level (leftmost) is more severe than that at a lower level. Thus, we decided to characterize the EC changes observed in release pairs by the following parameters: common prefix length, number of generalizations and specializations. Common prefix length refers to the number of levels that remained the same from left to right; the number of generalizations and specializations represent the number of deleted and added levels, respectively. Examples of EC changes described by our model are provided in Table S3 in Material S1.

To tackle the problem of analyzing and visualizing such a large amount of data representing the evolution of enzyme annotations across several releases of the UniProt/Swiss-Prot database, we proposed ADVISe [29]. It is a tool that provides a panoramic macro view of EC changes and presents further details on demand, such as the frequencies of change types segmented by the common prefix length, by the levels of generalization and specialization as well as by the UniProt/Swiss-Prot releases and by the enzyme families (leftmost EC level). Consequently, the trends of specialization, database growth and exceptions in which the EC numbers were deleted, divided or created and the revisions of past annotation errors can be identified using this tool. ADVISe also allows users to explore and compare the entry line types OC, RP and KW used in our supervised learning approach.

There are many reasons why an UniProt/Swiss-Prot entry has its EC annotation changed. In general, specializations in the EC annotation of an entry (for example entry P75289 that changed from 5.1.-.- to 5.1.3.4 from release 7 to 8 and entry P42404 that changed from 5.3.-.- to 5.3.1.27 from release 14 to 15) may be a result of increased evidence or they can indicate the creation of new EC numbers; generalizations in the EC annotation may be the result of misannotation (for example entry P17109 that changed from 2.5.1.64 to 2.5.1.- from release 13 to 14); an EC change that involves both specialization and generalization (for example entry P41407 that changed from 3.1.4.14 to 1.7.-.- from release 7 to 8) may be the result of a correction, where there was a misannotation and now there is enough evidence to make a correct and more precise annotation. Moreover, there are EC changes that result from changes in the EC classification system, as some EC numbers are deleted and others are created (for example, the EC number 2.5.1.64 was created in 2003 and deleted in 2008, when it was divided into 2.2.1.9 and 4.2.99.20). Trends in EC annotaion changes and their possible causes are further discussed in [29].

When we started this work in 2009, we collected a set of UniProt/Swiss-Prot releases and we noticed that there were some EC numbers with letter *n* followed by a number instead of a –. We searched UniProt/Swiss-Prot website and found that there are cases in which a partial EC number is used when the enzyme activity is already known because a complete EC number is not yet available in the EC number list from Nomenclature Committee of the International Union of Biochemistry and Molecular Biology (NC-IUBMB). Also, in some news from UniProt/Swiss-Prot [30], we found that the process of representing partial EC numbers associated to enzymes of already known activities using letter *n* with a preliminary EC number instead of *–* (for example 2.5.1.n1 instead of 2.5.1.-) was an ongoing process. We understood from this observation that there were enzymes of known activities that were yet represented as partial EC numbers using *–* and others that were already represented using *n*. So, we decided to consider partial EC numbers like 2.5.1.n1 as 2.5.1.-, which is in accordance with the NC-IUBMB.

#### Descriptive and predictive experiments

The data modeling was the same for the three types of experiments performed: Descriptive Multiclass, Predictive Multiclass and Predictive Common Source.

The training data from the EC changes and non-EC changes (also called the control set) are required to characterize and predict the EC number changes using a supervised learning approach. The algorithm needs to learn from these data in a training phase to be able to subsequently separate a set of entries that underwent EC changes from a set in which the EC annotations remained the same. For example, the entry with UniProt/Swiss-Prot id Q9PKH4, which underwent the EC change 3.1.3.2 to 3.1.3.5 from release 5 to 6, is an example of EC change type 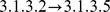, and the id P20611, for which the EC annotation remained the same from release 5 to 6, is an example of control set 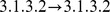.

Here, we proposed an occurrence data matrix to model the EC changes and non-changes. In such a matrix, the columns represent features (terms obtained from the OC, RP and KW line types), and the rows represent instances of the change or control set. The position 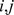 of this matrix is one whenever the instance of index *i* (a given entry) has the annotation attribute corresponding to the column of index *j*, and is zero otherwise. The last column represents the classes for each instance (row). The classes were modeled considering a source EC number (before the EC change) and a destination EC number (after the EC change), so an instance whose class is 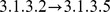 had its EC annotation changed from 3.1.3.2 to 3.1.3.5. A fragment of an occurrence matrix showing the EC change 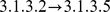, which occurred from release 5 to 6, and its control is provided in Table 1.

**Table 1:** Fragment of an occurrence matrix.

Id	F1	F2	F3	F4	F5	Class
Q8TUG3	1	1	0	1	0	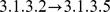
O67004	1	1	0	1	0	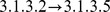
P34724	0	0	1	0	1	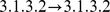
P44009	0	1	0	1	1	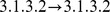

This fragment of an occurrence matrix shows the EC change 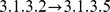, which occurred from release 5 to 6, and its control. F1 =  nucleotide-binding, F2 =  magnesium, F3 =  eukaryota, F4 =  metal-binding, F5 =  signal.

### Technique

#### Generation of occurrence matrix

To generate an occurrence data matrix to feed the supervised learning approach, for each type of EC change and for each release of the UniProt/Swiss-Prot in which such a change occurred, we parsed the text files of the entries that experienced the change and the files of the control group entries to extract the annotation attributes OC, RP and KW. We performed a text preprocessing on these data, which is a set of techniques applied in the text to reduce the data dimensionality and ambiguity. The following text preprocessing tasks were performed: *Normalization*, which is intended to remove punctuation from the text and convert the characters to lowercase; *Stop word removal*, which aims to remove stop words, which are extremely common words, such as pronouns and articles, and do not add information; *N-grams*, which is a contiguous sequence of *n* words from a given sequence of text that is used to capture some context present in the analyzed line types and to match not only exact terms but also approximate ones; *Stemming*, which is an algorithm that reduces inflected words to their stem, such as the words stem, stems and stemming, which have the root stem. The employed stemmer was a Java implementation of the Porter stemming algorithm [31], downloaded from the author's website [32]. We performed the same process for TrEMBL data set.

We processed the data from line types OC, RP and KW, which resulted in a set of features for the classification task. Considering a given type of change, the release in which this change occurred and an entry that underwent such a change, we extracted line types from this entry for all releases prior to the change (until the release immediately before the change).

#### EC change selection

As in the Descriptive Multiclass Experiment we employed a ten-fold cross-validation to evaluate the performance of our supervised learning approach, change types from UniProt/Swiss-Prot with at least ten examples were selected (discarded and used types of EC changes in each release are presented in Figure S3 in Material S1). The total number of EC change types was 1,968. Among them, 508 EC change types had at least 10 examples. Here, examples of the change types are 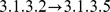 and 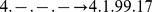. Q8TUG3 and O67004 are examples of entries that experienced change type 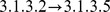.

In the TrEMBL data set, as it was used as test data for the Predictive Common Source Experiment, EC change types in which the common source (EC number before a change) was present in the training data of such experiment were selected. The total number of EC change types in TrEMBL data set was 471. Among them, 12 were present in the Predictive Common Source Experiment.

For some change types, such as 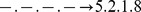 from release 39 to 40, there were many examples (288,932) in the control set, 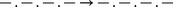, which represent entries that were not annotated with an EC in release 39 and remained without an EC annotation in release 40. Thus, we set an upper limit to the number of examples in the control set; otherwise, performing the tasks of dimensionality reduction (detailed in the next section) and classification would not be possible due to the computational cost and also, the training dataset would be extremely unbalanced. The upper limit chosen for the examples in the control set is the median of the number of EC change examples, which is 27. Additional information about this choice is provided in Figure S4 in Material S1.

#### Dimensionality reduction through SVD

Singular Value Decomposition (SVD) is a technique from linear algebra in which an *m* by *n* matrix *A* can be represented by the product 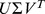 where *U* is an *m* by *m* matrix and its columns are the left singular vectors of *A*; 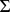 is an *m* by *n* diagonal matrix with its values in descending order; and *V* is an *n* by *n* matrix and its columns represents right singular vectors of *A*. To compress the data used in the classification task, reducing the number of features and noise, yet maintaining relevant semantic relationships among the terms, matrix *A* can be approximated by matrix 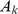 (with rank *k* where *k* is less than the rank of *A*) as: 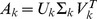.

To achieve 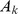, the first *k* singular values of *A* and their singular vectors were taken, and thus the resulting matrix has 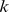 features: 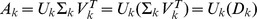. According to [33], 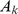 can be computed using only matrix 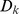, which is: 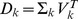.

In this work, the original matrix *A* was approximated by 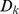. The same strategy for approximating 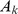 was adopted in [34], [35]. As stated by [36], the choice of *k* is an empirical matter; therefore approximations with *k* from 1 to 100 were generated, and the matrix that led to the best classification model was chosen. It is important to highlight that the applied dimensionality reduction via SVD may reduce the computational cost and memory requirements of the algorithms used in the classification task. SVD was used and discussed in a similar way in several studies [36]–[39].

#### Classification

In accordance with [40], classification is a supervised learning technique that consists of associating one or several predefined labels or classes with data objects. A classification model may be viewed as a function *f* that maps a set of attributes *x* to a given class *y*. The classification task is represented in Figure S5 in Material S1 and is performed as follows in each experiment: *Descriptive Multiclass Experiment*: This step aimed to verify whether the annotation attributes OC, RP and KW are able to discriminate entries that underwent a specific change in the EC number from those in which the EC annotation remained the same. We generated classification models using data matrices (constructed from the entire dataset, that is, the 44 UniProt/Swiss-Prot releases) that we reduced via SVD using *k* from 1 to 100, and we selected the best classification model. We evaluated the model performance through a ten-fold cross-validation. In addition to this experiment, we performed another one, using the same methodology, in which annotation attributes OC, RP and KW were used separately to discriminate entries that experienced a specific EC change from those which remained with the same EC annotation. It aimed to show the individual contribution of each line type.

*Predictive Multiclass Experiment*: We used EC change types previously modeled in the Descriptive Experiment to construct a classification model and predict the EC changes. Here, we reserved the last release in which a change type occurred to test the model. We consider as modeled EC change types those that had 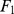 score greater than 0.5 (we detailed the 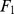 score in Section *Classifier evaluation strategy*). Only those were used because the change types that were not characterized in the Descriptive Experiment (in which the entire data set was used and a cross-validation was performed) are not expected to be predicted.

*Predictive Common Source Experiment*: We segmented the data set from the Predictive Multiclass (which comprises data from UniProt/Swiss-Prot) by the common source, and each source corresponds to a classifier. The common source here is the previous EC number (before the EC change) associated with an entry. For example, the EC changes 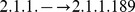, 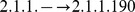 and their control 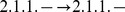 have the common source EC 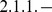, and there is one classifier in which the possible classes are these three EC changes. We performed this experiment expecting that making correct predictions using a more specialized classifier would be easier than the Predictive Multiclass Experiment in which a single classifier has 361 classes. Also, we performed a similar experiment using training data from UniProt/Swiss-Prot but test data from UniProt/TrEMBL expecting that the knowledge present in Swiss-Prot could be propagated to TrEMBL, improving the quality of its automatic annotations.

We employed and compared the classification algorithms Naïve Bayes [41], K Nearest Neighbor (KNN) [42] and C4.5, also called J48 [43]. We chose these algorithms due to their low memory requirements and short execution time.

#### Classifier evaluation strategy

We performed several experiments to choose the best classification model. We used the 100 matrices resulting from SVD with *k* (number of features or columns) varying from 1 to 100, and for each matrix, we applied three classification algorithms: Naïve Bayes, KNN with 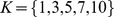 and J48. To assess the performance of the classifiers, we used the metrics 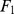 score (also called F measure) and Area Under the ROC Curve (AUC) [44].

The 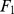 score is the harmonic mean of precision (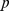) and recall (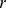), and it tends toward the least of these elements (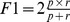). Precision is the fraction of actually positive instances among those that were predicted as positive by the classifier (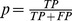) and recall refers to the fraction of actually positive instances that were retrieved by the classifier (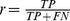).

The Receiver Operating Characteristic (ROC) Curve is a method to evaluate classifiers in which the true positive rate (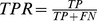) is plotted on the y axis and the false positive rate (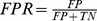) is plotted on the *x* axis. Some points of ROC curves have a well-defined interpretation: (FPR  = 1, TPR  = 0) means that all predictions are wrong, and (FPR  = 0, TPR  = 1) means that all positive and negative instances are correctly predicted. The case in which FPR  = 0 and TPR  = 1 is the ideal classifier, and the Area Under ROC Curve is 1. Thus, the closer AUC is to one, the better the model.

In the Descriptive Multiclass Experiment and the Predictive Multiclass Experiment, to select the best result for a specific classification algorithm, which is the matrix that led to this result, we applied a voting scheme. One vote was assigned for each result with the greatest value for 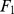 and similarly one vote was assigned for each result with the greatest value for AUC. Note that more than one result may present the maximum value for 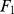 or AUC. If there was a tie, we chose the result obtained from the matrix with the smallest number of columns.

Similarly, after choosing the best result within a specific classification algorithm, we selected the best result among all techniques through the same voting scheme. In this case, if there was a tie, we chose the result with the best 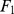. When comparing the results obtained from the different classification algorithms, those with similar AUC values may have quite different 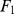 values (hence, different precision and recall). Therefore, we prioritize the best values of 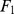 when there was a tie in the voting scheme.

In the Predictive Common Source Experiment, we chose the best result according to the best value for 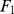 because in this experiment even classifiers with high values for AUC showed low values for 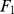 and therefore for precision and recall.

#### Implementation

SVD dimensionality reduction and all graphs were generated with R software [45], version 2.10.1. We implemented the data collection and processing in Java Development Kit 6 and performed the classification task using algorithms from Weka Data Mining Software [46] version 3.6.2. The EC changes collected were stored in a MySql database, release 5.5.24.

## Results and Discussion

### Descriptive Multiclass Experiment

In this section, we present the results of the descriptive step. This experiment aimed to verify whether the line types OC, RP and KW are able to discriminate entries that experienced a specific change in their EC number from those that remained the same. We generated classification models using data matrices reduced via SVD with *k* from 1 to 100 and chose the best classification model as explained in Section *Classifier evaluation strategy*. We evaluated the model performance through a ten-fold cross-validation.

Table 2 provides the best result for this experiment. The complete results are provided in Tables S4, S5, S6, S7 in Material S1. Except for Naïve Bayes, the classifiers predicted the EC changes as their precision, recall and 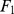 were approximately 70% and AUC was greater than 90%. We chose the KNN with 1 nearest neighbor as the best result due to its high 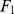 values, which was considered by our voting scheme. The KNN with 1 nearest neighbor indicates that, for each test instance considered in classification process, its nearest training instance is the most similar one and, therefore, helps to classify this test instance. As we try to use more neighbors (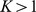), training instances from various different classes are considered, which increases the number of incorrect predictions. It is important to highlight that, in general, modeled classes (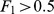) have more examples than unmodeled ones, as presented in Table S8 in Material S1.

**Table 2:** Best results for the Descriptive and Predictive Multiclass Experiments.

Multiclass experiment	Algorithm	# offeatures	FPR	Prec.	Rec.	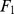	AUC
Descriptive	KNN_K1	38	0.01	0.74	0.74	0.74	0.95
Predictive	KNN_K1	13	0.08	0.41	0.32	0.25	0.65

In this table, # of features refers to the number of features or attributes (in the matrix that resulted in the best classification model). TPR corresponds to recall and was omitted.

In Table S9 in Material S1, the arithmetic and weighted means were calculated separately for the classes that represent EC changes (change set) and non-changes (control set). In general, the values were worse for the change set than the control set, which was expected because predicting an annotation that changes is more difficult than predicting an annotation that remains constant because the data set has more examples from the control set than the change set.

This experiment provided evidence that the annotation attributes OC, RP and KW are able to discriminate and characterize entries that experienced a specific EC number change because even in a multiclass classifier with 664 classes (a complex classification problem as the probability of correctly predicting a class at random is 1/664 or 0.15%), the values of 0.74 for 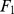 and 0.95 for AUC indicate that our classifier is far from random (when 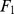 and AUC are approximately 0.5).

In addition to the Descriptive multiclass experiment, we performed another one, using the same methodology, in which annotation attributes OC, RP and KW were used separately to discriminate entries that experienced a specific EC change from those which remained with the same EC annotation. It aimed to show the individual contribution of each line type to predict EC number changes and we concluded that KW outperforms RP and OC. The results and discussions are presented in Tables S10 and S18 Material S1. We also conducted an experiment to assess whether changes in EC number annotation and KW line type occur at the same time and we concluded that although there is some correlation between EC and KW changes, for a significant amount of data they vary separately. This experiment and its results are detailed in Figure S6 and Tables S16 and S17 in Material S1.

### Predictive Experiments

The test data set was formed by the last occurrence of a certain type of EC change and the training data set comprised the previous occurrences of the same type of change. Consider the change 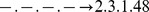, which occurred in releases 2, 6, 8, 9, 12, 14, 15, 43, and 44. We used data (line types RP, OC and KW) from releases 1, 5, 7, 8, 11, 13, 14 and 42 (which means that we collected the data before the change occurs) to train our classifier and we used data (RP, OC and KW) from release 43 to test our classifier (once again we collected data before the change occurs). Therefore, we observed what happened in the past and used the selected data as indicators of EC number changes, which means that data from the past are used to predict future events (in different releases and in different entries).

Here, we simulated a scenario in which all available information about a certain type of EC change was applied to predict an upcoming EC change of the same type, which means that our approach can predict only changes previously observed in the database.

#### Multiclass

The aim of the Predictive Multiclass Experiment was to make predictions for the last occurrence of each EC change type using a single multiclass classifier that comprises all possible classes. This experiment was performed similarly to the descriptive one, except for the EC change types, as here only those modeled in the Descriptive Multiclass Experiment were analyzed (361 classes).

The experimental results are provided in Table 2. The arithmetic and weighted means calculated separately for the change and control sets are shown in Table S13 in Material S1 (complete results are in Tables S11 and S12 in Material S1). The values of precision, recall, 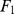 and AUC were significantly lower than those in the Descriptive Experiment. When the last release in which a change occurred was left for the test set, some examples were lost for the training set, which impacted in the quality of the result.

Therefore, to improve the results, we need more training examples or a more specialized classification task (with fewer classes than in the Predictive Multiclass Experiment). As we do not have control over the changes occurrence and amount, the changes were segmented by their common source, and a more specialized classification task was performed as detailed below.

#### Common source with Swiss-Prot test data

This experiment was performed as an attempt to improve the classification results of the Predictive Multiclass Experiment shown in Section *Multiclass*. The data set was segmented by the common EC source, and each source corresponds to a specific classifier. There are 24 common EC sources and thus 24 classifiers that are more specialized than the previous general multiclass, increasing the chance of making correct predictions (as there are fewer options of classes for each classifier). As explained in Section *Classifier evaluation strategy*, 100 matrices resulting from the SVD were processed by three classification algorithms: Naïve Bayes, KNN with 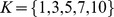 and J48. This process was performed for each of the 24 common source data sets, and the best results were chosen according to the best values for 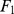.

The result of this experiment is provided in Table 3. The mean of the 24 best classifiers metrics was calculated to summarize the results (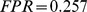, 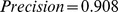, 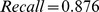, 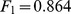, 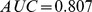). The mean had values of precision, recall and 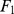 greater than 0.86.

**Table 3:** Results of the Common Source Experiment with Swiss-Prot test data.

Source	FPR	Prec.	Rec.	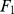	AUC	Algorithm	# of features	# of classes
-.-.-.-	0.10	0.66	0.34	0.31	0.66	KNN_K1	1	36
1.1.1.-	0.00	1.00	1.00	1.00	1.00	KNN_K1	11	2
1.10.2.2	0.00	1.00	1.00	1.00	1.00	KNN_K5	2	2
1.9.3.1	0.33	0.70	0.70	0.70	0.68	KNN_K10	2	2
2.-.-.-	0.31	0.77	0.42	0.32	0.62	N. Bayes	1	3
2.1.1.-	0.24	0.91	0.90	0.91	0.93	KNN_K7	74	3
2.3.1.-	0.96	0.93	0.96	0.95	0.91	KNN_K10	100	2
2.4.-.-	0.00	0.98	0.97	0.97	0.98	J48	13	2
2.7.1.-	0.03	0.93	0.88	0.89	0.89	KNN_K3	89	2
2.7.3.-	0.00	1.00	1.00	1.00	1.00	J48	30	2
2.7.7.48	0.30	0.70	0.66	0.66	0.55	KNN_K3	40	2
2.7.7.6	0.01	0.96	0.93	0.94	0.96	N. Bayes	32	2
3.-.-.-	0.01	0.95	0.90	0.91	0.94	KNN_K1	5	2
3.1.-.-	0.96	0.93	0.96	0.95	0.61	KNN_K1	100	2
3.1.13.-	0.06	0.95	0.95	0.95	0.91	KNN_K10	65	2
3.1.2.15	0.00	1.00	0.96	0.98	0.00	KNN_K10	100	2
3.2.1.18	0.93	0.87	0.93	0.90	0.50	J48	10	2
3.4.22.-	0.00	1.00	1.00	1.00	1.00	KNN_K10	100	2
3.4.25.-	0.33	1.00	1.00	1.00	0.97	KNN_K10	41	2
3.6.3.14	0.05	0.94	0.94	0.94	0.95	N. Bayes	12	2
4.2.2.-	0.64	0.80	0.72	0.62	0.80	KNN_K1	2	2
5.-.-.-	0.00	1.00	1.00	1.00	1.00	KNN_K1	4	2
6.-.-.-	0.90	0.81	0.90	0.85	0.50	N. Bayes	100	2
6.4.1.2	0.00	1.00	1.00	1.00	1.00	KNN_K1	10	2

Each line corresponds to the best result (classifier) obtained for each source as we used the training and test data from 1 up to 100 features after SVD processing and the classification techniques Naïve Bayes, J48 and KNN with 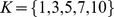. The last two columns refer to the number of features or attributes (in the occurrence matrix that resulted in the best classification model) and to the number of classes in each classifier. The TPR corresponds to the recall and was omitted.

In general, in this experiment classifiers have training datasets with many instances for change and control set. However, some of the classifiers in Table 3 (common sources 2.3.1.-, 3.1.-.-, 3.2.1.18, 4.2.2.- and 6.-.-.-) have a test dataset with many instances in the control set and very few instances in the change set. As an example, consider the common source 3.1.-.-. This source has two possible classes, 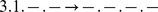 (change) and 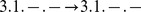 (control). In the training dataset, we have 78 instances in change set and 162 instances in control set. In the test dataset, we have 1 instance in the change set and 27 instances in the control set. In these cases (Table 3, common sources 2.3.1.-, 3.1.-.-, 3.2.1.18, 4.2.2.- and 6.-.-.-), if the classifier makes wrong predictions for all or almost all instances in the change set, which represents, indeed, just a few instances, it has a strong impact on FPR.

We observe that for common sources with enough number of test instances for control and change sets we obtained good results considering all metrics. Also, when we perform a ten fold cross validation (Descriptive experiment), which allows us to overcome the point of scarce number of test instances in change set, the classifier is able to predict changes and non changes. It strongly indicates that our classifiers (trained with enough number of control and change instances) are able to predict EC number changes and non changes and for those classifiers with high FPR, there is the limitation of the scarce number of test instances for change set. If we have more instances for the change set in the test dataset, these classifiers will be able to reach good performance, as they were already trained with enough number of control and change instances.

There was one common source, -.-.-.-, that had a significantly worse result compared with the mean. In addition, this origin had a high value of weight as it contains 36 types of changes and 2,631 instances of EC number changes. This common source is expected to have results worse than other common sources because it is composed of instances that do not have EC annotation and can receive any type of EC, which means that there are a lot more possible EC number changes in this classifier. It represents a difficult classification problem (with a great number of possible classes). Moreover, from the point of view of the semantic of common source -.-.-.-, it also represents a difficult classification problem, as this is composed by (i) entries that are not enzymes (and should stay -.-.-.-) (ii) entries for which it is not known if they are enzymes or not (it is not known if they should stay annotated as -.-.-.- or not), and (iii) entries which are enzymes but their classes are not known (there is a chance that they will be annotated with an EC number). Even so, we included the results of this common source in our work because we believe that in Table 3, the precision of 0.66 is relevant (it indicates that predicting a change is difficult, but if the classifier predicts an instance as a change, it has a considerable chance of being right). Finally, we decided to include the results of common source -.-.-.- because we understand that it is important to show and discuss negative and positive aspects and results related to the proposed strategy.

In Table S14 in Material S1, the arithmetic and weighted means were calculated separately for the change and control sets. In the weighted mean from the change set, the precision (0.756) is greater than the recall (0.274), which is also known as the true positive rate (TPR) or specificity. This result indicates that predicting a change is difficult, but if the classifier predicts an instance as a change, it has a great chance to make a correct prediction.

We would like to point out that enzyme classes received different attention over time. We performed a simple search for the names of higher level classes of EC hierarchy on February, 2012 in Google Scholar [47], PDB and PubMed [48] and calculated the percentage of results returned for each class in each of these three repositories. The results are shown in Table 4 and they indicate that some classes have been more studied than others over the years. The percentage of results for transferases is greater than 26% for all repositories. The percentage of results for hydrolases vary from 11% to 42%. On the other hand, the classes lyase and isomerase have percentage of results less than 10% in all repositories. So, the fact that certain EC classes have been more studied than others could have reflected in our work, as there are more examples of enzymes and their EC changes for classes extensively studied, which means that data are intrinsically biased. However, in the results of Table 3 we do not observe a relation between the quality of results and the EC classes as only EC change types with a minimun number of examples were considered in our strategy.

**Table 4:** Result of the search for higher level classes of EC number hierarchy.

EC Class	Scholar		PDB		PubMed	
	absolute value	(%)	absolute value	(%)	absolute value	(%)
oxidoreductase	122,000	6.5	7,731	1.8	499,969	20.2
transferase	942,000	50.0	10,897	26.5	712,758	28.8
hydrolase	215,000	11.4	16,054	39.1	1,040,771	42.1
lyase	154,000	8.2	3,202	7.8	118,865	4.8
isomerase	177,000	9.4	1,655	4.0	47,984	1.9
ligase	273,000	14.5	1,517	3.7	52,562	2.1

We performed a simple search for the names of higher level classes of EC number hierarchy on February, 2012 in repositories Google Scholar, PDB and PubMed (absolute value and percentage).

It is important to highlight that although some values of metrics seem low for the change set in Table S14 in Material S1, the data considered to be the correct answer (UniProt/Swiss-Prot EC annotations) can present some inconsistencies or even errors as we observed that changes in the EC annotation occur over time in this database. Furthermore, these metrics calculated from the Weka results do not take into consideration partial results (when not all predicted EC levels are correct). Thus, to provide a fair comparison between UniProt/Swiss-Prot and ENZYMAP, the predicted annotations were compared with the Swiss-Prot annotation considering from 1 to 4 levels of the EC number.

To extend this comparison, the DETECT tool [18] was used to make the EC predictions for the same Swiss-Prot entries used in our approach. Thus, predictions from the ENZYMAP, DETECT and Swiss-Prot annotations were compared. DETECT was chosen because it is a relatively new technique (2010) that is able to predict the EC number annotations based on global and local sequence alignments. It receives FASTA residue sequences separated by organism as input and then outputs EC number predictions. Although ENZYMAP and DETECT are essentially different (as ENZYMAP is based on entry line types OC, RP and KW from UniProt/Swiss-Prot and DETECT is based on residue sequence), their EC predictions can be used in a complementary manner to improve annotations.

#### Common source with TrEMBL test data

This experiment was conducted to demonstrate that ENZYMAP can be used to help to improve the quality of automatic annotations associated to enzyme data. For this reason UniProt/TrEMBL database was chosen as test data. We used training data from UniProt/Swiss-Prot (the same data set from previous Predictive Common Source Experiment) and test data from UniProt/TrEMBL, so, among the 471 EC change types from TrEMBL releases (43 and 44) we had to select those (12) that were present in the training data, totaling 1,247 test instances. There are 6 common EC sources and thus 6 classifiers.

The result of the experiment is provided in Table 5. The mean of the 6 best classifiers metrics was calculated to summarize the results (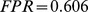, 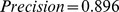, 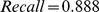, 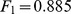, 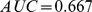). The values for precision, recall and 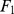 are greater than 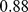. However, 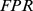 is considerably high, which happens because, for some EC sources, in the training dataset we have enough instances for change and control set, but in the test dataset, there are many instances in control set and very few instances for change set (EC sources 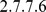, 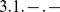 and 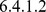 with 1, 2 and 1 examples of change respectively) and the classifier made a wrong prediction for all the instances in change set from these EC sources, which represents, indeed, very few instances. It had a strong impact on 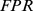.

**Table 5:** Results of the Common Source Experiment with TrEMBL test data.

Source	FPR	Prec.	Rec.	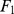	AUC	Algorithm	# of features	# of classes
-.-.-.-	0.13	0.82	0.68	0.74	0.80	N. Bayes	81	36
2.3.1.-	0.07	0.91	0.88	0.89	0.87	J48	5	2
2.7.7.6	0.96	0.93	0.96	0.95	0.50	KNN_K10	1	2
3.1.-.-	0.93	0.87	0.93	0.90	0.54	KNN_K1	100	2
3.6.3.14	0.58	0.92	0.91	0.89	0.79	N. Bayes	43	2
6.4.1.2	0.96	0.93	0.96	0.95	0.50	J48	10	2

Each line corresponds to the best result (classifier) obtained for each source as we used the training and test data from 1 up to 100 features after SVD processing and the classification techniques Naïve Bayes, J48 and KNN with 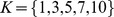. The last two columns refer to the number of features or attributes (in the occurrence matrix that resulted in the best classification model) and to the number of classes in each classifier. The TPR corresponds to the recall and was omitted.

In Table S15 in Material S1, the arithmetic and weighted means were calculated separately for the change and control sets. As already discussed in previous experiments, control set presents better results than change set. Nevertheless, in this experiment the weighted mean for change set presents excellent results, which is due to the high number of correct predictions for classes from change set which have many instances (for example (i) the class 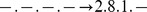, which has 705 instances and metrics 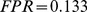, 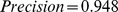, 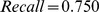, 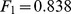, 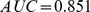 and (ii) the class 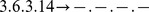, which has 169 instances and metrics 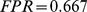, 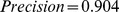, 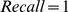, 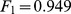, 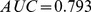). It means that, when a test set with many instances is provided, ENZYMAP is able to correctly predict EC number annotation changes.

### ENZYMAP, DETECT and Swiss-Prot comparison

**Figure 1:**
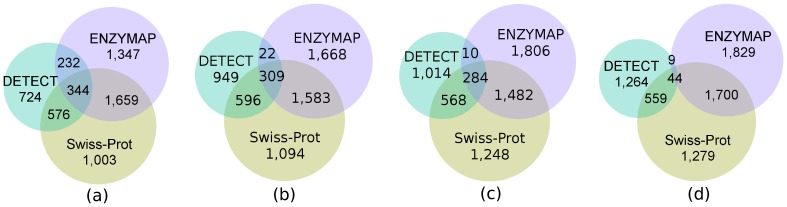
Comparison of ENZYMAP, DETECT and Swiss-Prot. We compared the EC number predictions made by ENZYMAP and DETECT and checked both against the UniProt/Swiss-Prot annotations. The number of predictions in which the techniques agree or disagree is presented in the diagrams. In (a), the first level of the EC number annotation is compared; In (b), (c) and (d), up to the second, third and fourth levels of the EC number annotation are compared.

The same input data set used for the predictive experiments in Section *Predictive experiments*, with 3,582 EC number changes, was given as input for DETECT 1.0. Our technique made 3,582 EC predictions, whereas DETECT made 1,876; both prediction sets were compared with the annotations from UniProt/Swiss-Prot. Figure 1 presents the comparison among the techniques.

For the first level shown in Figure 1 (a), 56% of the predictions made by ENZYMAP agree with UniProt/Swiss-Prot, whereas this rate is 49% for DETECT. If we consider the two approaches together, their intersection with UniProt/Swiss-Prot represents 72% of these database annotations, which shows that combining both of them increases the coverage of the annotations.

For levels 2, 3 and 4, the percentage of predictions made by ENZYMAP that are correct is greater than those made by DETECT, and both techniques together account for more than 64% of the database annotations as shown in Table 6. However, for level 4, the percentage of predictions made by DETECT that are correct decreases significantly and reaches 32%, whereas for ENZYMAP, the rate is 49%. Here, predictions that agree with the UniProt/Swiss-Prot are considered to be correct. The more specific the annotation, the more difficult it is to predict, which can lead to a common type of error called overprediction (when the annotation procedure assigns more levels than it should) [15]. Thus, in this aspect ENZYMAP outperforms DETECT.

**Table 6:** ENZYMAP and DETECT predictions that agree with UniProt/Swiss-Prot.

	Level 1	Level 2	Level 3	Level 4
ENZYMAP (%)	56	53	49	49
DETECT (%)	49	48	45	32
Coverage (%)	72	70	65	64

The rows ENZYMAP and DETECT respectively correspond to the percentage of predictions made by our approach and by DETECT that are in accordance with the UniProt/Swiss-Prot annotations. The Coverage represents the percentage of database annotations covered by the techniques used in a complementary manner. In this comparison we considered from 1 to 4 levels of EC number.

#### Case studies

In the common source 2.4.-.-, our technique predicted that entry Q5NDL2 should be annotated as 2.4.1.-. It was considered as an error because the Swiss-Prot annotation was 2.4.-.-. However, in release 2012_07 from July 2012 (released after our analysis), this entry received EC 2.4.1.255 in Swiss-Prot. We performed our prediction using the training data prior to release 2011_02 from February 2011 (inclusive) and the test data from release 2011_03 (March 2011), indicating that our technique anticipated the third EC level for entry Q5NDL2 16 months before it occurred in Swiss-Prot. DETECT did not return a result for this entry.

In this study we included multifunctional enzymes (which are those associated with more than one EC number). Despite being even more difficult for an automatic technique to predict EC number annotation for this kind of enzymes as they have characteristics related to different activities, ENZYMAP was able to predict that entries P48820 and P49792 (putative peptidyl-prolyl cis-trans isomerases from *Bos taurus* and *Homo sapiens* respectively), which were annotated only with EC number 5.2.1.8, would receive EC number 6.3.2.- in release 2012_05 from May, 2012 (their recommended name are now E3 SUMO-protein ligases RanBP2). DETECT predicted EC number 5.2.1.8 for entries P48820 and P49792, which is correct. However, ENZYMAP was able to predict an additional function to what were previously considered single function enzymes.

Entry Q5FWH2 was predicted to be 6.3.2.- for the test data from release 44, and this entry really experienced the change 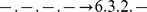 from release 43 to 44. In this case, our approach correctly predicted three EC levels starting from a non-annotated entry. DETECT did not return a result for Q5FWH2.

In the common source 3.2.1.18, 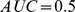 and 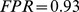, which seems a result worse than expected. However, in this case there were two classes, 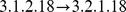 (with 27 test instances that were correctly predicted) and 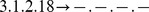 (with 2 test instances that were incorrectly predicted). We observe that all predictions for the class 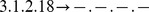, which means only 2 instances in the test release (14), were incorrect and that is why AUC and FPR are worse than expected.

In the common source 3.1.2.15, the values of the metrics were excellent, but 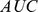 was zero. In this case, there were two classes, 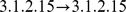 and 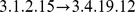. Predictions were made using the training data prior to release 2010_08 from July 2010 (inclusive) and the test data from release 2010_09 (August 2010). Among the 74 instances of change, 71 were correctly predicted. Nevertheless, in release 2010_09, there were no test instances in the control set (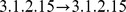), which explains why 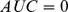.

DETECT and ENZYMAP predicted that entries O61694 and O94581, subunits of Cytochrome c oxidase of an insect and a yeast, respectively, should receive EC number 1.9.3.1, which refers to oxidoreductases acting on heme groups as electron donors and oxygen as acceptors. In UniProt/Swiss-Prot, an EC number is not assigned to these entries, indicating that they are not enzymes. The point is that Cytochrome c oxidase is a large transmembrane protein complex, with several subunits, which may introduce some ambiguity. The prediction is correct if we consider them to be part of the Cytochrome c oxidase enzymatic complex. However, these subunits (per se) may have no direct catalytic function. This case illustrates the difficulty of composing an unbiased annotation when the entry comes from multi-domain or multi-chain protein complexes with different functional units. Indeed, until release 15 (March 2009), Swiss-Prot assigned EC number 1.9.3.1 to these entries.

## Conclusion

In this work, we proposed ENZYMAP, a technique based on supervised learning to characterize and predict annotation changes in temporal data from UniProt/Swiss-Prot using entry line types that are already available in the database. Our proposal is intended to be an automatic complementary method (that can be used together with other techniques like the ones based on protein sequence and structure) that helps to improve the quality and reliability of enzyme annotations, suggesting possible corrections and anticipating annotation changes. Moreover, a common phenomenon in biological databases is that since a correction is made, this knowledge is not necessarily propagated to the whole database at once, but gradually and slowly. Our proposal can suggest corrections to database annotations, propagating the implicit knowledge for the whole dataset. To the best of our knowledge, there are no other works that propose this type of approach to improve the quality of biological annotations over time.

To characterize and predict the EC number changes, we performed three types of experiments: *Descriptive Multiclass*, in which we concluded that the selected line types (OC, RP and KW) were able to discriminate entries that experienced a specific change in the EC number from those that remained constant; *Predictive Multiclass*, which indicated that predicting the last occurrence of an EC change type using a multiclass classifier and having a scarce number of examples was not possible; and *Predictive Common Source*, which showed that predicting the last occurrence of an EC change type using more specialized classifiers even under the constraint of a scarce number of examples was possible. In addition, the predictions made by our proposal were compared with those made by the DETECT method, and both were checked against the Swiss-Prot annotations. The percentage of predictions made by ENZYMAP that were in accordance with Swiss-Prot was greater than the same percentage for DETECT for all 4 EC levels, and thus our technique outperformed DETECT in this aspect. Also, we conduct a Predictive Common Source experiment with test data from UniProt/TrEMBL in which we demonstrated that ENZYMAP can be used to improve the quality of automatic annotations associated to enzyme data.

As ENZYMAP hits annotation changes better than expected at random, it is identifying consistent recurring patterns in the training data, sufficient to support predictions that are not guesses. Our results indicate that line types may carry information sufficient to design a classifier able to make predictions of non-random trends in EC number annotation changes.

We envision a use case for ENZYMAP in which we employ the data available about EC changes in a set of UniProt/Swiss-Prot releases to predict upcoming changes, as performed in the Predictive Common Source Experiment. Thus, entries from the latest available release are given as input for a specific classifier trained with EC changes from all previous releases segmented by the common source, and this classifier returns EC predictions for each entry. As future work, we intend to investigate whether it is possible to assign a reliability score to our predictions to help the user decide whether s/he should accept this prediction. In addition, we are considering using Formal Concept Analysis to elucidate for domain experts what were the most relevant words among the different line types to make the predictions, an information that is lost due to SVD use.

As training data we used taxonomic information (OC), the extent of a reference relevant to an entry annotation (RP) and keywords (KW), which include a variety of biological information. For example, in the entry Q5FWH2, KW presents information such as metal-binding, structural motif (zinc-finger), biochemical process (Ubl conjugation) and subcellular localization (cytoplasm), among others. Nevertheless, also as future work, we plan to investigate whether there are other line types able to describe and predict changes in EC number annotation to be included in ENZYMAP. It is not just a matter of adding line types, as a large number of attributes will not necessarily improve predictions. If many irrelevant or redundant training data are included, it can have a negative impact on the results. So, if we add line types, we need to characterize and measure the dificulty of the new training dataset using, for example, entropy (related to the uncertainty of the data) and mutual information (concerning the attributes that carry the same information).

It is important to point out that the problem of changes in EC number annotations from Swiss-Prot is a real and relevant scenario. However, the technique and methodology developed in this work are independent of this application scenario and may be used in any scenario where there are large databases, which evolve in time and in which is relevant to identify and fix any errors or inconsistencies. For example, changes in functions of non-enzymes could be analysed using Gene Ontology (GO) [49] annotation, which is a controled and hierarchical vocabulary to represent gene and gene product attributes across species and databases.

## Supporting Information

Material S1 **Additional tables, graphs and details.** Tables, graphs and details about the used dataset, experiment design and complete results are provided in this document.
(PDF)
