# Supplementary material for: ENZYMAP: Exploiting Protein Annotation for Modeling and Predicting EC Number Changes in UniProt/Swiss-Prot
Source: PLoS One. 2014 Feb 19;9(2):e89162. doi: 10.1371/journal.pone.0089162 (PMC3929618; doi:10.1371/journal.pone.0089162)
Supplement: Material S1 — Additional tables, graphs and details. Tables, graphs and details about the used dataset, experiment design and complete results are provided in this document. (PDF) [file pone.0089162.s001.pdf]

ENZYMAP: Exploiting protein annotation for modeling and predicting EC number changes in UniProt/Swiss-Prot

Sabrina de Azevedo Silveira[1,2*], Raquel Cardoso de Melo-Minardi[1], Carlos Henrique da Silveira[3], Marcelo Matos Santoro[2], Wagner Meira Jr[1*]
1 Department of Computer Science, Universidade Federal de Minas Gerais, Belo Horizonte, Minas Gerais, Brazil
2 Department of Biochemistry and Immunology, Universidade Federal de Minas Gerais, Belo Horizonte, Minas Gerais, Brazil
3 Department of Biotechnology, Universidade Federal da Paraíba, João Pessoa, Paraíba, Brazil
∗ **E-mail: sabrinas@dcc.ufmg.br, meira@dcc.ufmg.br**

Supporting material S1

Availability: `www.dcc.ufmg.br/~sabrinas/enzymap`

Figure S1 - Statistics about UniProt/Swiss-Prot releases.

These graphs refer to Table S1. (a) Total number of entries and number of entries with EC number. (b) Percentage of entries with EC number.

(a)

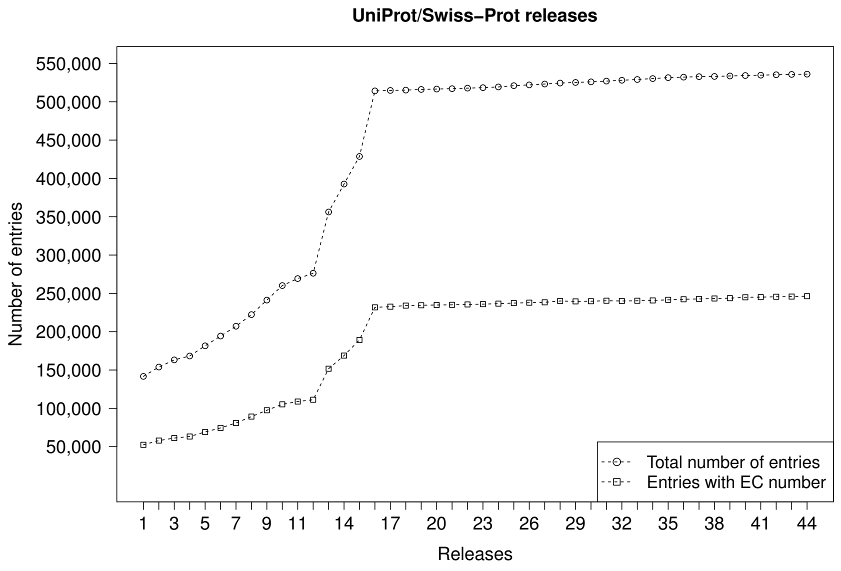
UniProt/Swiss–Prot releases
Number of entries
550,000
500,000
450,000
400,000
350,000
300,000
250,000
200,000
150,000
100,000
50,000
Total number of entries
Entries with EC number
1 3 5 7 9 11 14 17 20 23 26 29 32 35 38 41 44
Releases

(b)

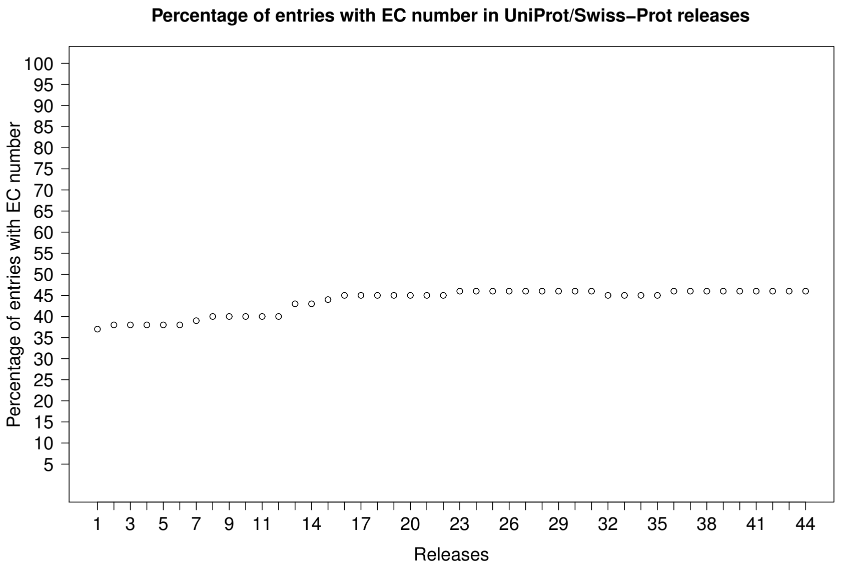
Percentage of entries with EC number in UniProt/Swiss–Prot releases
Percentage of entries with EC number
100 95 90 85 80 75 70 65 60 55 50 45 40 35 30 25 20 15 10 5
1 3 5 7 9 11 14 17 20 23 26 29 32 35 38 41 44
Releases

Figure S2 - Statistics about UniProt/Swiss-Prot pairs of releases.

These graphs refer to Table S2. (a) Number of entries in the set intersection of each release pair. (b) Percentage of entries in the set intersection of each release pair.

(a)

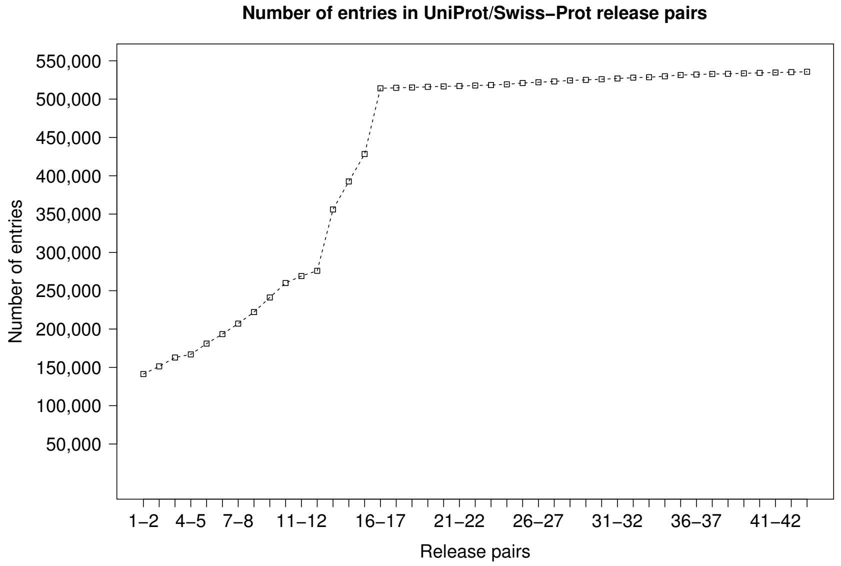

(b)

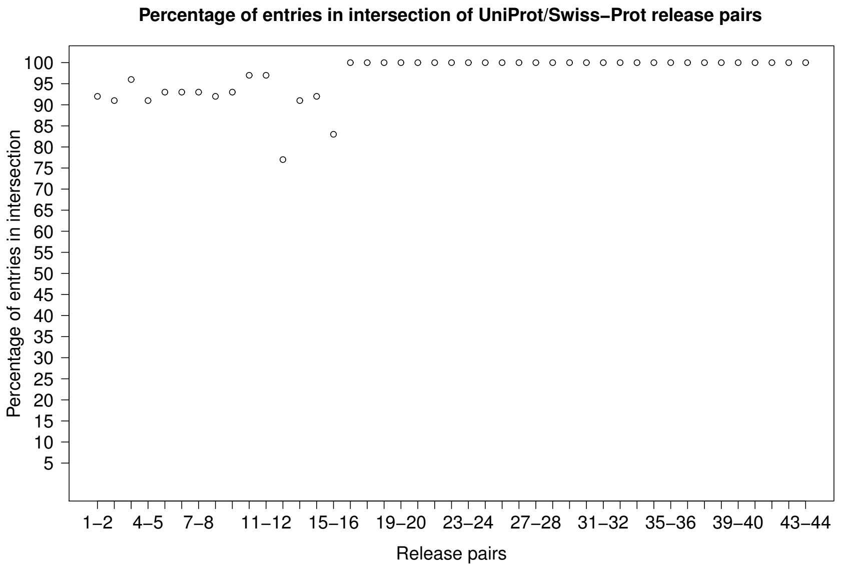

Figure S3 - Number of EC change types used and discarded.

EC change types with at least ten examples throughout all UniProt/Swiss-Prot releases were used in this study. Discarded and used types of EC changes in each release are presented in this figure.

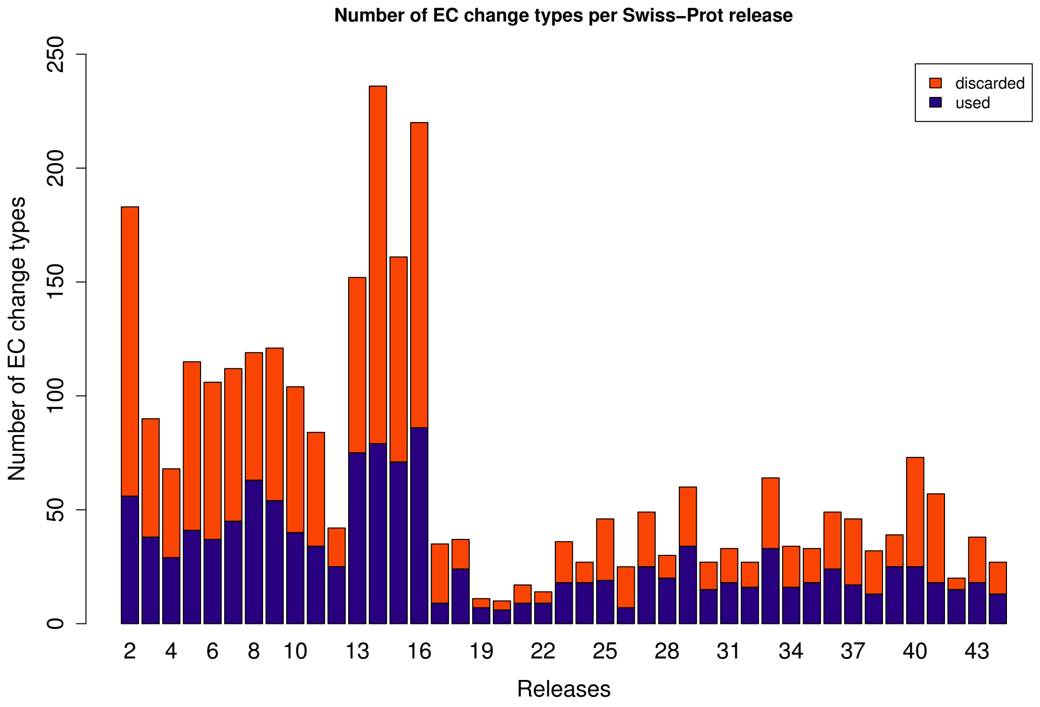

Figure S4 - Number of EC change examples for all EC change types.

The number of EC change examples is presented in x axis and the number of EC change types is presented in y axis. In (a) the histogram represents the number of EC change examples for all 508 EC change types; in (b) only changes with less than 200 examples are presented and in (c), changes with less than 100 examples. The upper limit chosen for instances in control set was 27, which is the median of the number of EC change examples. It is more representative for the number of examples than the mean, which is 102.2 with standard deviation 224.6.

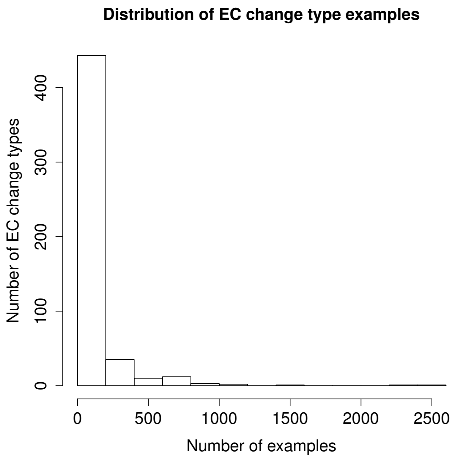
Distribution of EC change type examples
Number of EC change types
Number of examples
0
100
200
300
400
0
500
1000
1500
2000
2500

(a)

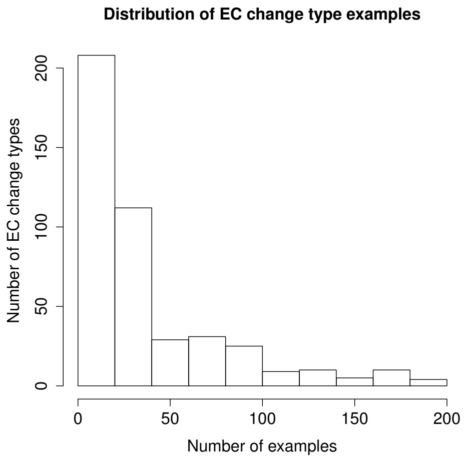
Distribution of EC change type examples
Number of EC change types
Number of examples
0
50
100
150
200
0
50
100
150
200

(b)

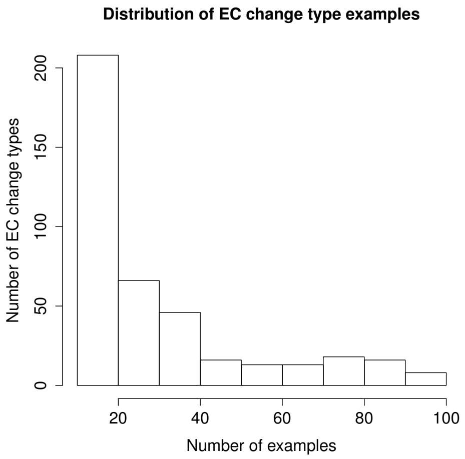
Distribution of EC change type examples
Number of EC change types
Number of examples
0
50
100
150
200
20
40
60
80
100

(c)

Figure S5 - Classification task flowchart.

This classification task flowchart represents the three types of experiments performed to characterize and predict the EC number changes: Descriptive multiclass, Predictive multiclass and Predictive common source.

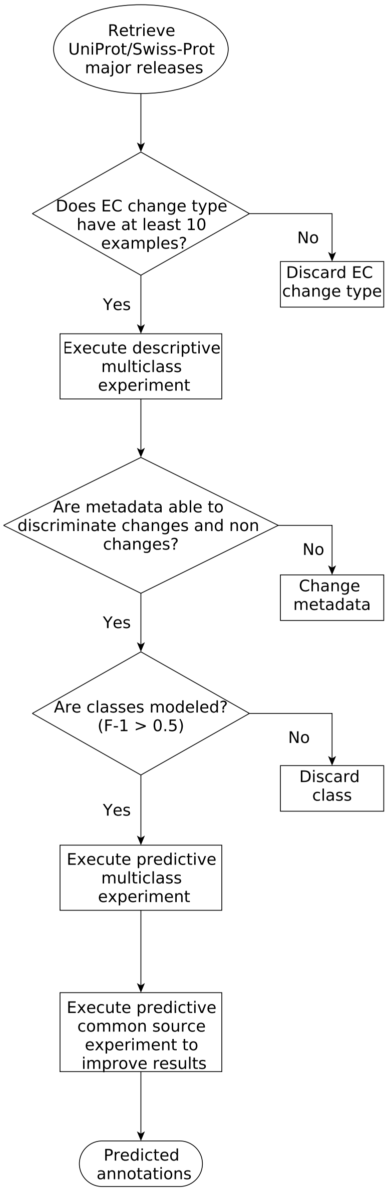

Figure S6 - Changes in KW line type and EC number.

The 44 releases of UniProt/Swiss-Prot were analyzed to check if changes in KW line type occur at the same time as changes in EC number annotation. An example of the data generated to perform this analysis is provided in Table S16. Orange represents instances in which EC number and KW changed at the same time; yellow indicates instances in which EC number and KW did not change (both stayed the same) and instances in which EC number and KW differ (one of them changed and the other did not) are depicted in black. A total of 18,727,155 records (or instances) of changes and non-changes were observed. Among those, there are 55,908 EC number changes and 1,074,763 KW changes. As the number of records in which neither EC number nor KW changed differs by orders of magnitude from the number of registers that represents change in EC or KW, random samples were obtained to perform a fair comparison. In (a), KW is used as reference, so all instances (1,074,763) in which KW line type changed were collected and a random sample of the same size was generated from instances in which KW did not change. In 1% of instances EC and KW changed at the same time (orange) and in 49% of instances EC and KW differed (depicted in black). In (b), EC is used as reference, so all instances (55,908) in which EC number changed were collected and a random sample of the same size was generated from instances in which EC number did not change. EC and KW changed at the same time in 23% of instances (orange), while in 30% of instances they differed (black). The quantitative results are presented in Table S17. These graphs indicate that a change in EC number implies in a change in KW, in Figure (b) in orange, more than a change in KW implies in a change in EC number, Figure (a) in orange. Figures (a) and (b) indicates that although there is some correlation between EC number and KW changes, in a significant amount of data they change separately (depicted in black).

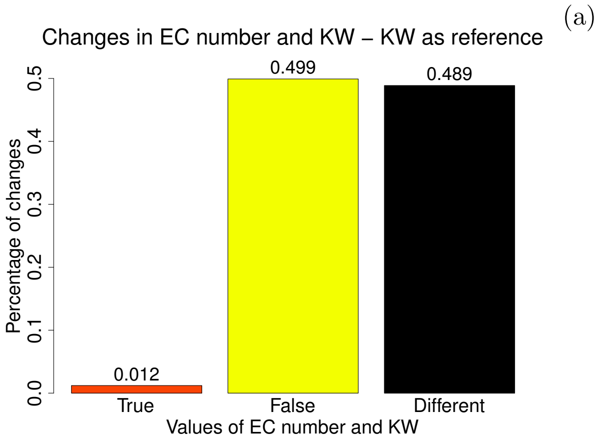

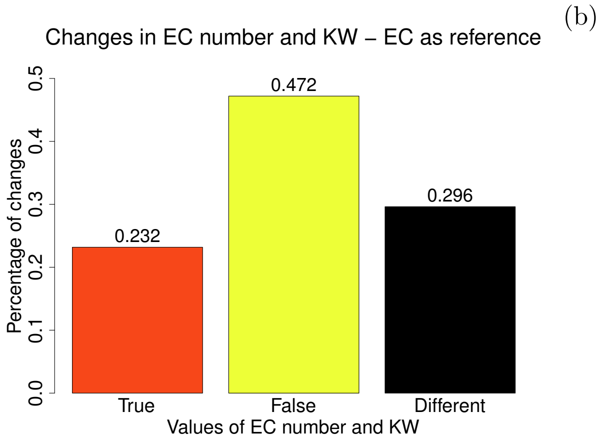

Table S1 - Releases 1 to 44 of UniProt/Swiss-Prot.

This table provides, for each release used in this study, the date in which the release was launched, percentage and number of entries with EC number and total number of entries.

Release index	Release name	Release date (MM/DD/YYYY)	% of entries with EC	Number of entries with EC	Total number of entries
1	1	12/15/2003	37	52,434	141,681
2	2	07/05/2004	38	57,931	153,871
3	3	10/25/2004	38	61,229	163,235
4	4	02/01/2005	38	63,221	168,297
5	5	05/10/2005	38	69,164	181,571
6	6	09/13/2005	38	74,468	194,317
7	7	02/07/2006	39	80,874	207,132
8	8	05/30/2006	40	89,245	222,289
9	9	10/31/2006	40	97,508	241,242
10	10	03/06/2007	40	105,225	260,175
11	11	05/29/2007	40	108,876	269,293
12	12	07/24/2007	40	111,230	276,256
13	13	02/26/2008	43	151,694	356,194
14	14	07/22/2008	43	168,849	392,667
15	15	03/24/2009	44	189,234	428,650
16	2010_01	01/19/2010	45	231,776	514,212
17	2010_02	02/09/2010	45	232,662	514,789
18	2010_03	03/02/2010	45	234,040	515,203
19	2010_04	03/23/2010	45	234,494	516,081
20	2010_05	04/20/2010	45	234,843	516,603
21	2010_06	05/18/2010	45	235,081	517,100
22	2010_07	06/15/2010	45	235,561	517,802
23	2010_08	07/13/2010	46	235,952	518,415
24	2010_09	08/10/2010	46	236,597	519,348
25	2010_10	10/05/2010	46	237,361	521,016
26	2010_11	11/02/2010	46	237,872	522,019
27	2010_12	11/30/2010	46	238,344	523,151
28	2011_01	01/11/2011	46	240,052	524,420
29	2011_02	02/08/2011	46	239,545	525,207
30	2011_03	03/08/2011	46	239,775	525,997
31	2011_04	04/05/2011	46	240,406	526,969
32	2011_05	05/03/2011	45	240,055	528,048
33	2011_06	05/31/2011	45	240,374	529,056
34	2011_07	06/28/2011	45	240,787	530,264
35	2011_08	07/27/2011	45	241,578	531,473
36	2011_09	09/21/2011	46	242,309	532,146
37	2011_10	10/19/2011	46	242,742	532,792
38	2011_11	11/16/2011	46	243,333	533,049
39	2011_12	12/14/2011	46	243,749	533,657
40	2012_01	01/25/2012	46	244,898	534,242
41	2012_02	02/22/2012	46	245,113	534,695
42	2012_03	03/21/2012	46	245,566	535,248
43	2012_04	04/18/2012	46	245,826	535,698
44	2012_05	05/16/2012	46	246,347	536,029

Table S2 - Release pairs and number of entries in the intersection.

This table provides the number of entries in the set intersection of each UniProt/Swiss-Prot release pair.

Release pair	Number of entries in $\cap$
1-2	141,249
2-3	151,318
3-4	162,812
4-5	166,933
5-6	181,005
6-7	193,382
7-8	207,069
8-9	222,181
9-10	241,189
10-11	260,065
11-12	269,152
12-13	276,011
13-14	356,036
14-15	392,597
15-16	428,331
16-17	514,121
17-18	514,740
18-19	515,180
19-20	516,049
20-21	516,593
21-22	517,045
22-23	517,769
23-24	518,350
24-25	519,302
25-26	521,007
26-27	522,001
27-28	523,101
28-29	524,367
29-30	525,107
30-31	525,960
31-32	526,934
32-33	528,024
33-34	528,573
34-35	529,826
35-36	531,443
36-37	532,076
37-38	532,780
38-39	533,028
39-40	533,643
40-41	534,227
41-42	534,678
42-43	535,207
43-44	535,682

Table S3 - Example of EC number changes across consecutive UniProt/Swiss-Prot releases and our prefix/generalization/specialization model.

Common prefix length refers to the number of levels that remained the same from left to right; the number of generalizations and specializations represent the number of deleted and added levels, respectively.

Previous EC number	Current EC number	UniProt id	Releases	Common prefix length	Degrees of generalization	Degrees of specialization
-.-.-.-	-.-.-.-	Q9K5T1	1-2	0	0	0
3.1.4.14	1.7.-.-	P41407	7-8	0	4	2
1.1.1.-	1.-.-.-	P52895	5-6	1	2	0
5.3.-.-	5.3.1.27	P42404	14-15	2	0	2
2.5.1.64	2.5.1.-	P17109	13-14	3	1	0
4.1.1.22	4.1.1.22	P95477	1-2	4	0	0

Text S1 - Descriptive Multiclass Experiment.

This experiment was performed in three different configurations regarding text preprocessing tasks n-grams and stemmer: (1) neither n-grams or stemmer were used; (2) just stemmer was used; (3) both n-grams and stemmer were used. The purpose of using these different configurations was to check which one was able to generate the best classification model and use the best configuration in subsequent predictive experiments.

The configuration with n-gram and without stemmer was not performed due to hardware constraints. As the occurrence matrix (detailed in Section *Generation of occurrence matrix* from our paper) for this configuration was the larger one (3.8 GB), the used machine ran out of RAM memory. This matrix is large because stemmer technique, which would reduce the number of features mapping inflected words to their stem, was not applied.

The results are presented in Tables S4 (Neither n-grams or stemmer were used), S5 (just stemmer was used) and S6 (both n-grams and stemmer were used). Table S7 summarizes the results. The configuration (3), in which both n-gram and stemmer were applied, is slightly better than the others, thus in predictive experiments this was the chosen configuration.

Table S4 - Results for configuration 1: occurrence matrix generated using neither n-grams or stemmer.

Technique	Votes	Maximum	Features	TPR	FPR	Precision	Recall	F_1	AUC
Naïve Bayes	1	TPR	93	0.494	0.004	0.672	0.494	0.526	0.927
	0	FPR	1	0.255	0.255	0.065	0.255	0.104	0.715
	1	Precision	93	0.494	0.004	0.672	0.494	0.526	0.927
	1	Recall	93	0.494	0.004	0.672	0.494	0.526	0.927
	1	F_1	93	0.494	0.004	0.672	0.494	0.526	0.927
	**1**	**AUC**	**82**	**0.481**	**0.004**	**0.662**	**0.481**	**0.511**	**0.928**
KNN_K1	**2**	**TPR**	**99**	**0.741**	**0.005**	**0.74**	**0.741**	**0.738**	**0.952**
	0	FPR	1	0.559	0.008	0.545	0.559	0.55	0.901
	**2**	**Precision**	**99**	**0.741**	**0.005**	**0.74**	**0.741**	**0.738**	**0.952**
	**2**	**Recall**	**99**	**0.741**	**0.005**	**0.74**	**0.741**	**0.738**	**0.952**
	**2**	F_1	**99**	**0.741**	**0.005**	**0.74**	**0.741**	**0.738**	**0.952**
	1	AUC	94	0.74	0.005	0.739	0.74	0.737	0.952
KNN_K3	**2**	**TPR**	**90**	**0.713**	**0.009**	**0.705**	**0.713**	**0.703**	**0.963**
	0	FPR	1	0.487	0.017	0.458	0.487	0.466	0.887
	1	Precision	97	0.712	0.009	0.705	0.712	0.702	0.963
	**2**	**Recall**	**90**	**0.713**	**0.009**	**0.705**	**0.713**	**0.703**	**0.963**
	**2**	F_1	**90**	**0.713**	**0.009**	**0.705**	**0.713**	**0.703**	**0.963**
	1	AUC	97	0.712	0.009	0.705	0.712	0.702	0.963
KNN_K5	0	TPR	100	0.701	0.013	0.684	0.701	0.683	0.965
	0	FPR	1	0.46	0.024	0.41	0.46	0.428	0.879
	0	Precision	100	0.701	0.013	0.684	0.701	0.683	0.965
	0	Recall	100	0.701	0.013	0.684	0.701	0.683	0.965
	**2**	F_1	**95**	**0.701**	**0.013**	**0.683**	**0.701**	**0.684**	**0.966**
	**2**	**AUC**	**95**	**0.701**	**0.013**	**0.683**	**0.701**	**0.684**	**0.966**
KNN_K7	1	TPR	48	0.691	0.015	0.667	0.691	0.669	0.966
		FPR	1	0.44	0.031	0.376	0.44	0.4	0.873
	1	Precision	64	0.691	0.016	0.669	0.691	0.669	0.966
	1	Recall	48	0.691	0.015	0.667	0.691	0.669	0.966
	**2**	F_1	**55**	**0.691**	**0.016**	**0.667**	**0.691**	**0.67**	**0.966**
	1	AUC	79	0.689	0.016	0.666	0.689	0.667	0.966
KNN_K10	1	TPR	54	0.676	0.020	0.644	0.676	0.648	0.967
		FPR	1	0.419	0.040	0.341	0.419	0.369	0.866
		Precision	86	0.676	0.022	0.647	0.676	0.647	0.966
	1	Recall	54	0.676	0.020	0.644	0.676	0.648	0.967
	**2**	F_1	**21**	**0.676**	**0.018**	**0.640**	**0.676**	**0.649**	**0.967**
	1	AUC	46	0.675	0.020	0.642	0.675	0.647	0.967
J48	**2**	**TPR**	**88**	**0.744**	**0.006**	**0.732**	**0.744**	**0.733**	**0.937**
		FPR	1	0.498	0.014	0.468	0.498	0.479	0.831
	1	Precision	90	0.743	0.006	0.732	0.743	0.732	0.937
	**2**	**Recall**	**88**	**0.744**	**0.006**	**0.732**	**0.744**	**0.733**	**0.937**
	**2**	F_1	**88**	**0.744**	**0.006**	**0.732**	**0.744**	**0.733**	**0.937**
	1	AUC	85	0.743	0.006	0.731	0.743	0.732	0.937

Table S5 - Results for configuration 2: occurrence matrix generated using just stemmer.

Technique	Votes	Maximum	Features	TPR	FPR	Precision	Recall	F_1	AUC
Naïve Bayes	0	TPR	99	0.492	0.004	0.67	0.492	0.523	0.927
	0	FPR	1	0.255	0.255	0.065	0.255	0.104	0.715
	0	Precision	94	0.491	0.004	0.671	0.491	0.523	0.927
	1	Recall	99	0.492	0.004	0.67	0.492	0.523	0.927
	1	F_1	100	0.492	0.004	0.671	0.492	0.524	0.926
	**0**	**AUC**	**89**	**0.491**	**0.004**	**0.671**	**0.491**	**0.523**	**0.928**
KNN_K1	1	TPR	97	0.741	0.005	0.739	0.741	0.737	0.952
	0	FPR	1	0.559	0.008	0.546	0.559	0.551	0.901
	1	Precision	92	0.74	0.005	0.739	0.74	0.737	0.952
	1	Recall	97	0.741	0.005	0.739	0.741	0.737	0.952
	**2**	F_1	**98**	**0.741**	**0.005**	**0.739**	**0.741**	**0.738**	**0.952**
	1	AUC	82	0.739	0.005	0.738	0.739	0.736	0.952
KNN_K3	**2**	**TPR**	**90**	**0.713**	**0.009**	**0.706**	**0.713**	**0.703**	**0.963**
	0	FPR	1	0.486	0.016	0.457	0.486	0.465	0.887
	**2**	**Precision**	**90**	**0.713**	**0.009**	**0.706**	**0.713**	**0.703**	**0.963**
	**2**	**Recall**	**90**	**0.713**	**0.009**	**0.706**	**0.713**	**0.703**	**0.963**
	**2**	F_1	**90**	**0.713**	**0.009**	**0.706**	**0.713**	**0.703**	**0.963**
	1	AUC	84	0.712	0.009	0.705	0.712	0.702	0.963
KNN_K5	1	TPR	91	0.701	0.013	0.683	0.701	0.683	0.966
	0	FPR	1	0.46	0.023	0.411	0.46	0.429	0.879
	1	Precision	95	0.701	0.013	0.684	0.701	0.684	0.966
	1	Recall	91	0.701	0.013	0.683	0.701	0.683	0.966
	**1**	F_1	**48**	**0.701**	**0.012**	**0.683**	**0.701**	**0.685**	**0.965**
	1	AUC	91	0.701	0.013	0.683	0.701	0.683	0.966
KNN_K7	1	TPR	53	0.691	0.016	0.666	0.691	0.669	0.966
	0	FPR	1	0.441	0.03	0.378	0.441	0.401	0.874
	1	Precision	100	0.691	0.017	0.668	0.691	0.668	0.966
	1	Recall	53	0.691	0.016	0.666	0.691	0.669	0.966
	**2**	F_1	**55**	**0.691**	**0.016**	**0.667**	**0.691**	**0.67**	**0.966**
	1	AUC	53	0.691	0.016	0.666	0.691	0.669	0.966
KNN_K10	0	TPR	86	0.677	0.022	0.646	0.677	0.647	0.966
	0	FPR	1	0.419	0.040	0.341	0.419	0.369	0.866
	0	Precision	85	0.676	0.022	0.647	0.676	0.647	0.966
	0	Recall	86	0.677	0.022	0.646	0.677	0.647	0.966
	**1**	F_1	**22**	**0.676**	**0.018**	**0.64**	**0.676**	**0.649**	**0.967**
	1	AUC	48	0.676	0.020	0.643	0.676	0.648	0.968
J48	1	TPR	90	0.742	0.006	0.731	0.742	0.731	0.936
	0	FPR	1	0.498	0.013	0.469	0.498	0.479	0.831
	1	Precision	90	0.742	0.006	0.731	0.742	0.731	0.936
	1	Recall	90	0.742	0.006	0.731	0.742	0.731	0.936
	1	F_1	90	0.742	0.006	0.731	0.742	0.731	0.936
	**1**	**AUC**	**61**	**0.741**	**0.006**	**0.729**	**0.741**	**0.729**	**0.937**

Table S6 - Results for configuration 3: occurrence matrix generated using both n-grams and stemmer.

Technique	Votes	Maximum	Features	TPR	FPR	Precision	Recall	F_1	AUC
Naïve Bayes	**2**	**TPR**	**97**	**0.507**	**0.005**	**0.672**	**0.507**	**0.534**	**0.929**
	0	FPR	1	0.255	0.255	0.065	0.255	0.104	0.715
	1	Precision	100	0.505	0.005	0.672	0.505	0.532	0.929
	**2**	**Recall**	**97**	**0.507**	**0.005**	**0.672**	**0.507**	**0.534**	**0.929**
	**2**	F_1	**97**	**0.507**	**0.005**	**0.672**	**0.507**	**0.534**	**0.929**
	1	AUC	90	0.499	0.004	0.667	0.499	0.525	0.929
KNN_K1	1	TPR	95	0.744	0.005	0.741	0.744	0.74	0.952
	0	FPR	1	0.567	0.008	0.554	0.567	0.559	0.903
	1	Precision	97	0.744	0.005	0.742	0.744	0.74	0.952
	1	Recall	95	0.744	0.005	0.741	0.744	0.74	0.952
	1	F_1	95	0.744	0.005	0.741	0.744	0.74	0.952
	**1**	**AUC**	**38**	**0.741**	**0.005**	**0.739**	**0.741**	**0.738**	**0.953**
KNN_K3	1	TPR	29	0.718	0.009	0.709	0.718	0.709	0.962
	0	FPR	1	0.495	0.016	0.467	0.495	0.475	0.891
	**2**	**Precision**	**100**	**0.718**	**0.009**	**0.712**	**0.718**	**0.709**	**0.963**
	1	Recall	29	0.718	0.009	0.709	0.718	0.709	0.962
	1	F_1	29	0.718	0.009	0.709	0.718	0.709	0.962
	1	AUC	86	0.716	0.009	0.709	0.716	0.707	0.963
KNN_K5	1	TPR	95	0.711	0.013	0.696	0.711	0.695	0.966
	0	FPR	1	0.468	0.024	0.421	0.468	0.438	0.884
	**2**	**Precision**	**100**	**0.711**	**0.013**	**0.697**	**0.711**	**0.696**	**0.966**
	1	Recall	95	0.711	0.013	0.696	0.711	0.695	0.966
	**2**	F_1	**100**	**0.711**	**0.013**	**0.697**	**0.711**	**0.696**	**0.966**
	1	AUC	95	0.711	0.013	0.696	0.711	0.695	0.966
KNN_K7	**2**	**TPR**	**96**	**0.702**	**0.016**	**0.683**	**0.702**	**0.682**	**0.966**
	0	FPR	1	0.449	0.03	0.387	0.449	0.41	0.88
	**2**	**Precision**	**96**	**0.702**	**0.016**	**0.683**	**0.702**	**0.682**	**0.966**
	**2**	**Recall**	**96**	**0.702**	**0.016**	**0.683**	**0.702**	**0.682**	**0.966**
	**2**	F_1	**96**	**0.702**	**0.016**	**0.683**	**0.702**	**0.682**	**0.966**
	1	AUC	83	0.701	0.017	0.68	0.701	0.68	0.966
KNN_K10	**1**	**TPR**	**81**	**0.691**	**0.022**	**0.664**	**0.691**	**0.664**	**0.966**
	0	FPR	1	0.426	0.040	0.35	0.426	0.377	0.873
	1	Precision	97	0.689	0.022	0.665	0.689	0.663	0.967
	**1**	**Recall**	**81**	**0.691**	**0.022**	**0.664**	**0.691**	**0.664**	**0.966**
	**1**	F_1	**81**	**0.691**	**0.022**	**0.664**	**0.691**	**0.664**	**0.966**
	1	AUC	97	0.689	0.022	0.665	0.689	0.663	0.967
J48	**2**	**TPR**	**88**	**0.738**	**0.006**	**0.728**	**0.738**	**0.727**	**0.934**
	0	FPR	1	0.505	0.014	0.473	0.505	0.484	0.839
	2	Precision	97	0.738	0.006	0.73	0.738	0.727	0.934
	**2**	**Recall**	**88**	**0.738**	**0.006**	**0.728**	**0.738**	**0.727**	**0.934**
	**2**	F_1	**88**	**0.738**	**0.006**	**0.728**	**0.738**	**0.727**	**0.934**
	2	AUC	88	0.738	0.006	0.728	0.738	0.727	0.934

Table S7 - Best performance of EC change prediction for each classification technique separated by configuration.

(1) neither n-grams or stemmer were used; (2) just stemmer was used; (3) both n-grams and stemmer were used. Configuration (3) was selected as the best one and KNN_K1 with 38 features was choosen as the best result.

Experiment	Votes	Technique	Feature	TPR	FPR	Precision	Recall	F_1	AUC
**1**	0	Naïve Bayes	82	0.481	0.004	0.662	0.481	0.511	0.928
	**1**	**KNN_K1**	**99**	**0.741**	**0.005**	**0.740**	**0.741**	**0.738**	**0.952**
	0	KNN_K3	90	0.713	0.009	0.705	0.713	0.703	0.963
	0	KNN_K5	95	0.701	0.013	0.683	0.701	0.684	0.966
	0	KNN_K7	55	0.691	0.016	0.667	0.691	0.67	0.966
	1	KNN_K10	21	0.676	0.018	0.640	0.676	0.649	0.967
	0	J48	88	0.744	0.006	0.732	0.744	0.733	0.937
**2**	0	Naïve Bayes	89	0.491	0.004	0.671	0.491	0.523	0.928
	**1**	**KNN_K1**	**98**	**0.741**	**0.005**	**0.739**	**0.741**	**0.738**	**0.952**
	0	KNN_K3	90	0.713	0.009	0.706	0.713	0.703	0.963
	0	KNN_K5	48	0.701	0.012	0.683	0.701	0.685	0.965
	0	KNN_K7	55	0.691	0.016	0.667	0.691	0.67	0.966
	1	KNN_K10	22	0.676	0.018	0.640	0.676	0.649	0.967
	0	J48	61	0.741	0.006	0.729	0.741	0.729	0.937
**3**	0	Naïve Bayes	97	0.507	0.005	0.672	0.507	0.534	0.929
	**1**	**KNN_K1**	**38**	**0.741**	**0.005**	**0.739**	**0.741**	**0.738**	**0.953**
	0	KNN_K3	100	0.718	0.009	0.712	0.718	0.709	0.963
	1	KNN_K5	100	0.711	0.013	0.697	0.711	0.696	0.966
	1	KNN_K7	96	0.702	0.016	0.683	0.702	0.682	0.966
	1	KNN_K10	81	0.691	0.022	0.664	0.691	0.664	0.966
	0	J48	88	0.738	0.006	0.728	0.738	0.727	0.934

Table S8 - Statistics of Descriptive multiclass experiment best result.

Best result of Descriptive multiclass experiment (KNN_K1 with 38 features and configuration 3 in which both n-grams and stemmer were used). This table provides the mean, standard deviation, median and total of instances for all classes and for classes that represent control and change set. The last column shows the number of classes in general and separated by control and change set. The statistics are divided in modeled (classes that have $F_1 > 0.5$) and unmodeled (classes that have $F_1 <= 0.5$).

	Class	Mean	Standard deviation	Median	Total of instances	Number of classes
Modeled	All	183.1	1155.8	37	63,540	347
	Control	292.6	2119.7	34	28,972	99
	Change	139.4	286.3	37	34,568	248
Unmodeled	All	61.2	123.6	23	19,414	317
	Control	36.1	48.4	27	2,059	57
	Change	66.8	134.0	21	17,355	260

Table S9 - Arithmetic and weighted mean for the best result of descriptive multiclass experiment (KNN_K1 with 38 features).

The means were calculated separately for classes that represent EC changes (Change Set) and non changes (Control Set).

	Control Set		Change Set	
Metrics	Arithmetic Mean	Weighted Mean	Arithmetic Mean	Weighted Mean
TPR	0.549	0.879	0.511	0.659
FPR	0.000	0.010	0.000	0.002
Precision	0.592	0.864	0.529	0.664
Recall	0.549	0.879	0.511	0.659
F_1	0.564	0.870	0.515	0.659
AUC	0.892	0.969	0.893	0.942

Table S10 - Descriptive multiclass experiment for line types OC, RP and KW used separately.

Line types (metadata) RP, OC, and KW were used separately to characterize EC changes. Here the individual contribution of each line type is shown.

Experiment	Votes	Technique	Feature	TPR	FPR	Precision	Recall	F_1	AUC
OC	0	Naive Bayes	1	0.118	0.066	0.097	0.118	0.102	0.615
	**1**	**KNN_K1**	**64**	**0.275**	**0.208**	**0.139**	**0.275**	**0.146**	**0.777**
	1	KNN_K3	92	0.274	0.208	0.130	0.274	0.144	0.778
	0	KNN_K5	77	0.273	0.209	0.124	0.273	0.143	0.777
	0	KNN_K7	67	0.273	0.209	0.123	0.273	0.142	0.777
	0	KNN_K10	59	0.273	0.210	0.118	0.273	0.140	0.776
	0	J48	9	0.275	0.222	0.128	0.275	0.139	0.764
RP	0	Naive Bayes	1	0.143	0.100	0.083	0.143	0.104	0.634
	**1**	**KNN_K1**	**64**	**0.277**	**0.220**	**0.167**	**0.277**	**0.146**	**0.733**
	1	KNN_K3	72	0.272	0.223	0.137	0.272	0.138	0.735
	1	KNN_K5	97	0.271	0.225	0.133	0.271	0.135	0.735
	1	KNN_K7	84	0.270	0.227	0.128	0.270	0.133	0.735
	1	KNN_K10	99	0.269	0.229	0.126	0.269	0.130	0.735
	0	J48	16	0.273	0.221	0.136	0.273	0.138	0.717
KW	0	Naive Bayes	34	0.503	0.004	0.675	0.503	0.526	0.925
	**1**	**KNN_K1**	**80**	**0.787**	**0.012**	**0.767**	**0.787**	**0.760**	**0.990**
	1	KNN_K3	99	0.776	0.013	0.748	0.776	0.746	0.991
	0	KNN_K5	100	0.769	0.015	0.737	0.769	0.736	0.990
	0	KNN_K7	100	0.763	0.016	0.729	0.763	0.728	0.990
	0	KNN_K10	94	0.753	0.018	0.715	0.753	0.715	0.990
	0	J48	81	0.772	0.012	0.743	0.772	0.742	0.971

Table S11 - Predictive experiment: multiclass with train and test data.

The last release in which a certain type of EC change occurs is used as test data and previous releases are used as train data.

Technique	Votes	Maximum	Features	TPR	FPR	Precision	Recall	F_1	AUC
Naïve Bayes	0	TPR	100	0.201	0.064	0.320	0.201	0.214	0.699
	0	FPR	92	0.176	0.066	0.323	0.176	0.184	0.698
	0	Precision	53	0.150	0.019	0.387	0.150	0.191	0.685
	0	Recall	100	0.201	0.064	0.320	0.201	0.214	0.699
	**1**	F_1	**65**	**0.200**	**0.039**	**0.344**	**0.200**	**0.236**	**0.692**
	1	AUC	74	0.184	0.056	0.328	0.184	0.208	0.704
KNN_K1	0	TPR	34	0.318	0.089	0.338	0.318	0.236	0.646
	0	FPR	22	0.314	0.102	0.387	0.314	0.225	0.639
	0	Precision	1	0.239	0.013	0.564	0.239	0.243	0.657
	0	Recall	34	0.318	0.089	0.338	0.318	0.236	0.646
	**1**	F_1	**13**	**0.316**	**0.075**	**0.406**	**0.316**	**0.247**	**0.652**
	1	AUC	60	0.316	0.085	0.399	0.316	0.240	0.663
KNN_K3	1	TPR	57	0.301	0.084	0.488	0.301	0.242	0.634
	0	FPR	25	0.282	0.105	0.287	0.282	0.204	0.611
	0	Precision	54	0.298	0.077	0.498	0.298	0.241	0.649
	1	Recall	57	0.301	0.084	0.488	0.301	0.242	0.634
	1	F_1	57	0.301	0.084	0.488	0.301	0.242	0.634
	**1**	**AUC**	**12**	**0.283**	**0.066**	**0.399**	**0.283**	**0.232**	**0.657**
KNN_K5	0	TPR	17	0.283	0.088	0.386	0.283	0.213	0.622
	0	FPR	28	0.269	0.112	0.199	0.269	0.194	0.624
	0	Precision	93	0.267	0.081	0.506	0.267	0.220	0.643
	0	Recall	17	0.283	0.088	0.386	0.283	0.213	0.622
	**1**	F_1	**57**	**0.282**	**0.086**	**0.502**	**0.282**	**0.231**	**0.635**
	1	AUC	75	0.269	0.076	0.449	0.269	0.226	0.657
KNN_K7	0	TPR	56	0.272	0.090	0.503	0.272	0.218	0.641
	0	FPR	26	0.259	0.114	0.202	0.259	0.184	0.629
	0	Precision	91	0.260	0.077	0.510	0.260	0.216	0.650
	0	Recall	56	0.272	0.090	0.503	0.272	0.218	0.641
	**2**	F_1	**13**	**0.260**	**0.049**	**0.238**	**0.260**	**0.225**	**0.671**
	2	AUC	13	0.260	0.049	0.238	0.260	0.225	0.671
KNN_K10	**2**	**TPR**	**100**	**0.270**	**0.085**	**0.497**	**0.270**	**0.225**	**0.666**
	0	FPR	26	0.251	0.107	0.201	0.251	0.182	0.637
	0	Precision	69	0.257	0.079	0.515	0.257	0.212	0.647
	2	Recall	100	0.270	0.085	0.497	0.270	0.225	0.666
	2	F_1	100	0.270	0.085	0.497	0.270	0.225	0.666
	2	AUC	100	0.270	0.085	0.497	0.270	0.225	0.666
J48	0	TPR	90	0.310	0.079	0.383	0.310	0.254	0.607
	0	FPR	32	0.300	0.115	0.301	0.300	0.219	0.669
	0	Precision	44	0.302	0.073	0.688	0.302	0.248	0.621
	0	Recall	90	0.310	0.079	0.383	0.310	0.254	0.607
	1	F_1	46	0.299	0.052	0.418	0.299	0.255	0.638
	**1**	**AUC**	**16**	**0.296**	**0.084**	**0.249**	**0.296**	**0.221**	**0.692**

Table S12 - Predictive multiclass experiment with train and test data: best performance of EC change prediction for each classification algorithm.

Number of Votes	Algorithm	Number of Features	TPR	FPR	Precision	Recall	F_1	AUC
1	Naive Bayes	65	0.200	0.039	0.344	0.200	0.236	0.692
**1**	**KNN_K1**	**13**	**0.316**	**0.075**	**0.406**	**0.316**	**0.247**	**0.652**
0	KNN_K3	12	0.283	0.066	0.399	0.283	0.232	0.657
0	KNN_K5	57	0.282	0.086	0.502	0.282	0.231	0.635
0	KNN_K7	13	0.260	0.049	0.238	0.260	0.225	0.671
0	KNN_K10	100	0.270	0.085	0.497	0.270	0.225	0.666
1	J48	16	0.296	0.084	0.249	0.296	0.221	0.692

Table S13 - Arithmetic and weighted mean for the best result of predictive multiclass experiment (KNN_K1 with 13 features).

The means were calculated separately for classes that represent EC changes (Change Set) and non changes (Control Set).

	Control Set		Change Set	
Metrics	Arithmetic Mean	Weighted Mean	Arithmetic Mean	Weighted Mean
TPR	0.515	0.828	0.092	0.255
FPR	0.016	0.229	0.001	0.002
Precision	0.585	0.524	0.114	0.269
Recall	0.515	0.828	0.092	0.255
F_1	0.512	0.605	0.078	0.188
AUC	0.804	0.826	0.641	0.721

Table S14 - Arithmetic and weighted mean for the best result of common source experiment with Swiss-Prot test data.

The means were calculated separately for classes that represent EC changes (Change Set) and non changes (Control Set).

	Control Set		Change Set	
Metrics	Arithmetic Mean	Weighted Mean	Arithmetic Mean	Weighted Mean
TPR	0.881	0.908	0.269	0.274
FPR	0.287	0.301	0.038	0.070
Precision	0.855	0.741	0.287	0.756
Recall	0.881	0.908	0.269	0.274
F_1	0.859	0.806	0.249	0.293
AUC	0.812	0.825	0.687	0.643

Table S15 - Arithmetic and weighted mean for the best result of common source experiment with TrEMBL test data.

The means were calculated separately for classes that represent EC changes (Change Set) and non changes (Control Set).

	Control Set		Change Set	
Metrics	Arithmetic Mean	Weighted Mean	Arithmetic Mean	Weighted Mean
TPR	0.778	0.648	0.229	0.773
FPR	0.525	0.338	0.091	0.227
Precision	0.888	0.680	0.218	0.903
Recall	0.778	0.648	0.229	0.773
F_1	0.803	0.649	0.221	0.829
AUC	0.647	0.666	0.609	0.826

Table S16 - Fragment of table generated to study KW and EC number changes.

This is a fragment of a table with 18,727,155 lines, each one representing an observation of EC number and KW line type changes and non-changes for the 44 UniProt/Swiss-Prot releases studied in pairs. The 44 releases of UniProt/Swiss-Prot were analyzed to check if changes in KW line type occur at the same time as changes in EC number annotation. Column *EC Change* has value 1 if the EC number of the correspondent entry changed in the specific release pair and this column has value 0 otherwise. The same applies to *KW Change* column. The whole table is available in the ENZYMAP website.

Id	Release Pair	Old EC	New EC	EC Change	KW Change
P47997	1-2	2.7.1.-	2.7.1.-	0	0
Q09815	1-2	2.7.1.-	2.7.1.37	1	0
Q479B1	14-15	1.4.99.1	1.4.99.1	0	0
A5VHH9	14-15	-.-.-.-	2.1.1.-	1	1
B7NFT7	43-44	3.6.1.22	3.6.1.22	0	0
C6A2P7	43-44	-.-.-.-	2.7.7.77	1	1

Table S17 - Comparison of changes in KW line type and EC number.

Results of changes in KW and EC number. An example of the data generated to perform this analysis is provided in Table S16. Column *EC=KW=0* represents instances in which EC number and KW did not change; column *EC=KW=1* refers to instances in which EC number and KW changed at the same time; column *EC=KW* shows instances in which EC and KW changed at the same time or both stayed the same and column *EC≠KW* represents instances whose EC and KW changed separately (one of them is 0 and the other is 1). In the row *Percentage over the dataset* the absolute values from each column is divided by the number of instances of the reference dataset, while in the row *Percentage over changes or non-changes* values are divide by half of the number of instances from the reference dataset. In (a), KW is used as reference, so all instances (1,074,763) in which KW line type changed were collected and a random sample of the same size was generated from instances in which KW did not change. In (b), EC is used as reference, so all instances (55,908) in which EC number changed were collected and a random sample of the same size was generated from instances in which EC number did not change.

	Percentage	EC=KW=0	EC=KW=1	EC=KW	EC≠KW
(a)	Over the dataset	0.499	0.012	0.511	0.489
	Over changes or non-changes	0.998	0.024	-	-

	Percentage	EC=KW=0	EC=KW=1	EC=KW	EC≠KW
(b)	Over the dataset	0.472	0.232	0.704	0.296
	Over changes or non-changes	0.944	0.464	-	-

Table S18 - OC, RP and KW used separately.

The Descriptive multiclass experiment with OC, RP and KW used separately aimed to show the individual contribution of line types OC, RP and KW to discriminate entries that underwent a specific change in the EC number from those in which the EC annotation remained the same. The methodology is the same used in the Descriptive Multiclass Experiment, the only difference is that three classification models were generated from three data matrices, one for each line type.

Table S18 provides the best results for each line type. The complete results are in the Supporting material S1, Table S10, which shows the best result for each classification algorithm. The line type RP is slightly better than OC to characterize changes in EC annotation and KW outperforms OC and RP. KW is potencially good to characterize EC changes as it is a controlled vocabulary which summarises the content of an entry. KW is automatically assigned in TrEMBL and manually verified in Swiss-Prot manual curation process. Also, we conducted an experiment using the complete dataset (44 releases of UniProt/Swiss-Prot) to assess whether changes in EC number annotation and KW line type occur at the same time and we concluded that although there is some correlation between EC and KW changes, for a significant amount of data they vary separately: when EC is used as reference, KW only changes simultaneously for 23% of the instances, whereas when KW is used as reference, EC changes concomitantly for only 1%. This finding strongly indicates that KW and EC changes are not always coupled. This experiment and its results are detailed in Supporting material S1, Figure S6 and Tables S16 and S17.

Results showed in Table S18 provide evidence that some UniProt line types are better than others to characterize EC number changes. Moreover, it is important to point out that the multiclass classifier with 664 classes based on KW was able to identify consistent recurring patterns in the training data as its results (0.76 for F_1) are much better than expected at random (the probability of correctly predicting a class at random is 1/664 or 0.15%)

Descriptive experiment	Algorithm	# of features	FPR	Prec.	Rec.	F_1	AUC
OC	KNN_K1	64	0.208	0.139	0.275	0.146	0.777
RP	KNN_K1	64	0.220	0.167	0.277	0.146	0.733
KW	KNN_K1	80	0.012	0.767	0.787	0.760	0.990
